# Plasma Small RNAs as Predictive and Monitoring Biomarkers for Combination Immunotherapy in Advanced Gastric Cancer

**DOI:** 10.1002/cam4.71339

**Published:** 2025-11-04

**Authors:** Jingshuai Fang, Yan Sun, Yuhui Yu, Zheng Fu, Yitong Tian, Yizhang Chen, Fen Guo, Jie Tang, Caiwang Yan, Xi Chen, Xiaofeng Chen, Guangfu Jin

**Affiliations:** ^1^ School of Biological Science and Medical Engineering Southeast University Nanjing China; ^2^ Department of Epidemiology, School of Public Health Nanjing Medical University Nanjing China; ^3^ State Key Laboratory Cultivation Base of Biomarkers for Cancer Precision Prevention and Treatment, Collaborative Innovation Center for Cancer Personalized Medicine Nanjing Medical University Nanjing China; ^4^ Department of Medical Oncology, Jiangsu Cancer Hospital and Jiangsu Institute of Cancer Research The Affiliated Cancer Hospital of Nanjing Medical University Nanjing China; ^5^ Nanjing Drum Tower Hospital Center of Molecular Diagnostic and Therapy, Chinese Academy of Medical Sciences Research Unit of Extracellular RNA, State Key Laboratory of Pharmaceutical Biotechnology, Jiangsu Engineering Research Center for MicroRNA Biology and Biotechnology, NJU Advanced Institute of Life Sciences (NAILS), Institute of Artificial Intelligence Biomedicine School of Life Sciences, Nanjing University Nanjing China; ^6^ Department of Oncology The First Affiliated Hospital with Nanjing Medical University Nanjing China; ^7^ Department of Oncology The Affiliated Wuxi People's Hospital of Nanjing Medical University; Wuxi Medical Center Wuxi China; ^8^ Department of Oncology Suzhou Hospital of Nanjing Medical University Suzhou China; ^9^ Department of Medical Oncology Liyang People Hospital Liyang China; ^10^ Gastric Cancer Center The First Affiliated Hospital with Nanjing Medical University Nanjing China

**Keywords:** advanced gastric cancer, immunotherapy, small RNA, tumor biomarker

## Abstract

**Background:**

Immunotherapy has become a new standard treatment for advanced gastric cancer (aGC). However, current biomarkers are insufficient for accurately identifying true responders, emphasizing the need for novel biomarkers.

**Methods:**

Between December, 2020, and October, 2023, we recruited 91 consecutive aGC patients (49 in the discovery and 42 in the validation cohorts). Plasma samples were collected at baseline and after two cycles of immunotherapy. We conducted small RNA (sRNA) next‐generation sequencing on 140 samples. Additionally, we investigated previously reported potential biomarkers, including PD‐L1 combined positive score (CPS), inflammation scores, and serological tumor biomarkers.

**Results:**

In the discovery cohort, we identified two pre‐treatment sRNAs significantly associated with response to immunotherapy: high levels of hsa‐miR‐3916 (*p =* 0.020) and low levels of hsa‐miR‐181d‐5p (*p =* 0.046), confirmed in the validation cohort (*p* = 0.011 and *p* = 0.013, respectively). The AUCs for predicting response using these two sRNAs were 0.77 (95% CI; 0.62–0.93) and 0.83 (95% CI; 0.71–0.96), respectively. When integrating PD‐L1 CPS with these two sRNAs, the AUCs were 0.82 (95% CI; 0.68–0.96) and 0.83 (95% CI; 0.70–0.97) for the discovery and validation cohorts, respectively. Furthermore, when combining PD‐L1 CPS and serological tumor biomarkers with these two sRNAs, the AUCs were 0.89 (95% CI; 0.79–1.00) and 0.83 (95% CI; 0.70–0.96) for the discovery and validation cohorts, respectively. After combination immunotherapy, responders exhibited decreased levels of hsa‐miR‐320c (*p* = 0.006) and increased levels of hsa‐miR‐26b‐5p (*p* = 0.007). Additionally, patients with decreased hsa‐miR‐320c demonstrated a trend towards improved PFS (median: 9.17 vs. 3.03 months, *p* < 0.001) and OS (median: 16.43 vs. 10.23 months, *p* = 0.115).

**Conclusion:**

These findings provide valuable insights into the sRNA features associated with response to combination immunotherapy in aGC patients and suggest potential biomarkers useful for selecting patients likely to benefit from immunotherapy.

## 1 Introduction

Gastric cancer (GC) ranks as the fifth most common cancer worldwide and is a leading cause of cancer‐related death [1]. The majority of GC patients are diagnosed at an advanced stage, and advanced gastric cancer (aGC) is typically considered incurable, with a 5‐year survival rate of approximately 4% [2, 3]. Palliative chemotherapy remains the standard of care for aGC, but immunotherapy has revolutionized cancer treatment, yielding significant clinical responses in GC. Findings from the ATTRACTION‐4 [4] and CHECKMATE 649 [5] trials suggest that the combination of nivolumab and chemotherapy may be more effective than chemotherapy alone. In contrast, various trials involving pembrolizumab or nivolumab have shown variable response rates (10%–26%) in GC within the salvage setting, with no selective biomarker or PD‐L1 combined positive score (CPS) positivity greater than or equal to 1% [6, 7, 8].

To date, microsatellite instability‐high (MSI‐H) [9, 10, 11], Epstein–Barr virus (EBV) [9], and tumor mutation burden (TMB) [12, 13] are currently under evaluation as biomarkers for immunotherapy. However, significant intra‐patient heterogeneity weakens the reliability of these tests [14]. Additionally, some patients may lack adequate tumor tissue for PD‐L1 expression, MSI, and EBV testing. Recently, inflammation scores such as the platelet‐to‐lymphocyte ratio (PLR), neutrophil‐to‐lymphocyte ratio (NLR), and systemic immune‐inflammation index (SII), along with conventional serological tumor biomarkers like carcinoembryonic antigen (CEA), carbohydrate antigen (CA) 72‐4, CA125, CA19‐9, and alpha‐fetoprotein (AFP), have been evaluated as non‐invasive biomarkers for immunotherapy response [15, 16, 17]. Nevertheless, these inflammation scores remain limited in achieving robust and consistent performance in clinical practice.

Small RNAs (sRNAs) are defined as polymeric ribonucleic acid molecules less than 200 nucleotides in length, including microRNA (miRNA), tRNA‐derived small RNA (tsRNA), PIWI‐interacting RNA (piRNA), and small interfering RNA (siRNA), among others [18]. As crucial transcriptional regulators, sRNAs perform a variety of essential functions within cells, including cell proliferation, differentiation [19], apoptosis [20], organ development [21], and particularly in tumorigenesis [22]. Recently, the potential use of circulating sRNAs in plasma and other body fluids for cancer screening and prognosis has emerged [23, 24, 25]. Furthermore, studies have indicated that sRNAs may serve as promising biomarkers for predicting the efficacy of immunotherapy in advanced non‐small cell lung cancers (NSCLCs) [25]. Despite these findings, a comprehensive global profile of plasma sRNA features associated with sensitivity to immunotherapy in GC patients remains to be elucidated.

In this study, we conducted sRNA sequencing on 140 plasma samples from 91 aGC patients who were treated with combination immunotherapy. Our objective was to identify plasma sRNAs as potential biomarkers for predicting and dynamically monitoring the efficacy of combination immunotherapy.

## 2 Methods

### 2.1 Study Design

**FIGURE 1:**
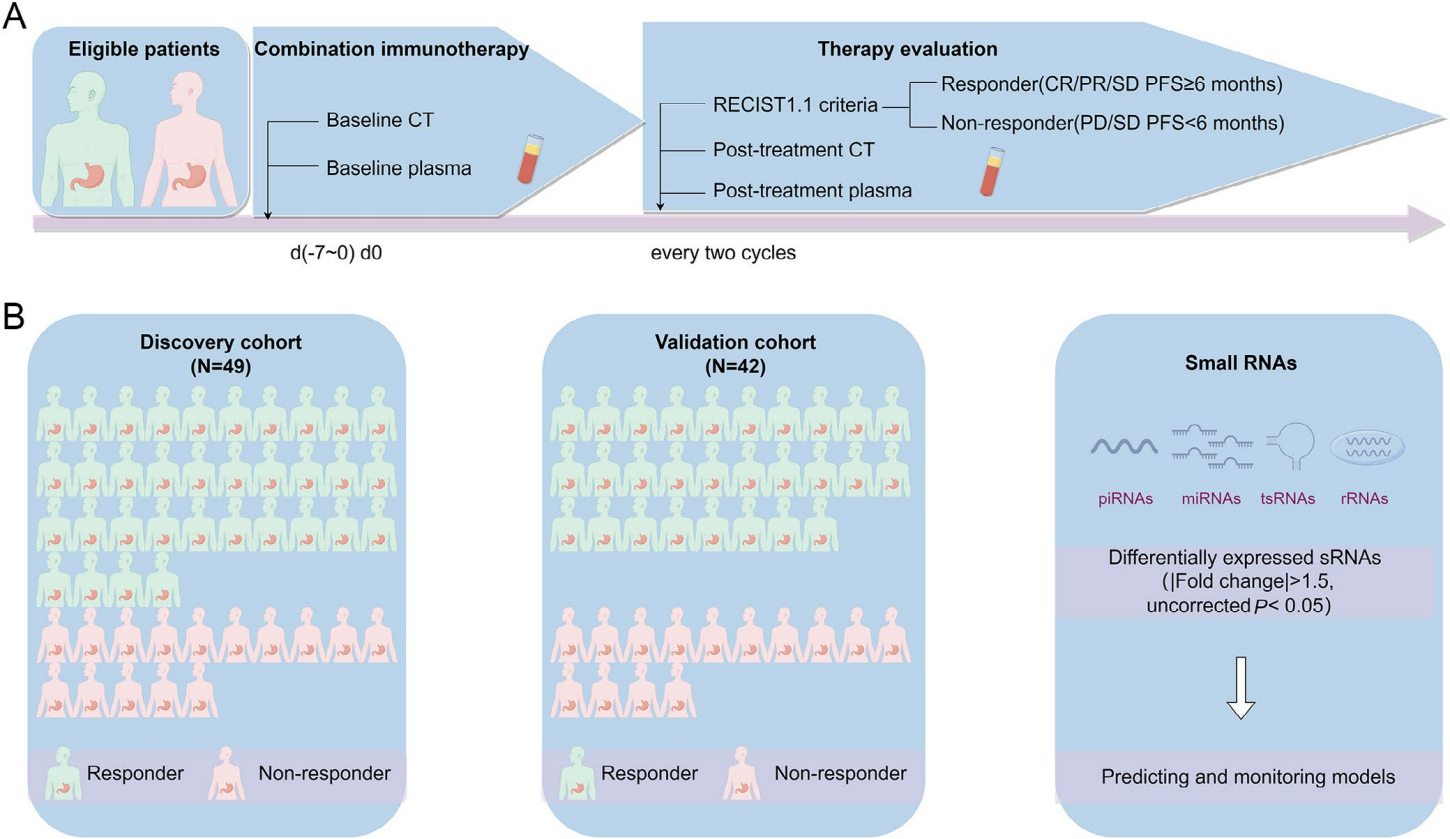
Study design. (A) Baseline plasma samples from patients with advanced gastric cancer were collected prior to the initiation of combination immunotherapy. Tumor status was evaluated by computed tomography (CT) every two cycles. Based on RECIST 1.1 criteria, patients were classified as responders (complete response (CR), partial response (PR), or stable disease (SD) with progression‐free survival (PFS) of ≥ 6 months) and non‐responders (SD with PFS ≤ 6 months or progressive disease (PD)). Post‐treatment plasma samples were collected at each evaluation point. (B) The discovery and validation cohorts included 49 and 42 patients, respectively, whose plasma samples underwent small RNA sequencing.

The current study analyzed a total of 140 plasma samples, comprising 98 samples from 49 aGC patients in the discovery cohort and 42 samples from 42 aGC patients in the validation cohort (Figure 1A). Small RNA sequencing analyses were performed on these 140 plasma samples from both cohorts.

### 2.2 Participants in the Discovery and Validation Cohort

Between December 2, 2020, and October 10, 2023, we recruited 91 consecutive aGC patients who had measurable, histologically confirmed metastatic and/or recurrent gastric adenocarcinomas from The First Affiliated Hospital of Nanjing Medical University [26] and The Affiliated Cancer Hospital of Nanjing Medical University. To be eligible for participation in this study, patients had to meet the following criteria: (1) a histologically confirmed diagnosis of gastric adenocarcinoma; (2) willingness to consent to the collection of EDTA‐anticoagulated whole blood for biomarker analysis at baseline and after two cycles of immunotherapy; (3) at least one measurable lesion according to Response Evaluation Criteria in Solid Tumors (RECIST) 1.1 [27]; (4) adequate organ function as per protocol; and (5) an Eastern Cooperative Oncology Group (ECOG) performance status (PS) of 0 or 1. Furthermore, all patients had not previously received treatment with anti‐PD‐1, anti‐PD‐L1, or anti‐PD‐L2 antibodies.

The enrolled patients received one of the immune checkpoint inhibitors (ICIs), including Nivolumab, Tislelizumab, and Camrelizumab, in combination with chemotherapy. The chemotherapy regimen consisted of one or more of the following agents: oxaliplatin, fluorouracil, capecitabine, albumin‐bound paclitaxel, irinotecan, and trastuzumab. Additionally, some patients received Anlotinib or Apatinib, which are VEGFR‐2 inhibitors, concurrently.

Tumor responses were evaluated by computed tomography (CT) every two cycles from the initiation of treatment until disease progression, according to RECIST 1.1 criteria. Patients with complete response (CR), partial response (PR), or stable disease (SD) with progression‐free survival (PFS) of ≥ 6 months were categorized as responders (R). In contrast, those with SD and PFS < 6 months or progressive disease (PD) were classified as non‐responders (NR) at the best overall response (BOR) evaluation. In the discovery cohort of 49 patients, 34 were identified as responders and 15 as non‐responders, with all patients providing paired baseline and post‐treatment plasma samples. In the validation cohort of 42 patients, 28 were classified as responders and 14 as non‐responders, with baseline plasma samples available for all patients (Figure 1B).

### 2.3 PD‐L1 Status Classification

Formalin‐fixed, paraffin‐embedded tissue specimens were subjected to immunohistochemistry (IHC) utilizing the 22C3 pharmDx assay (Agilent Technologies, USA). The expression of PD‐L1 protein was quantified using the CPS, which is the proportion of PD‐L1 positive cells (including tumor cells, lymphocytes, and macrophages) in the total viable tumor cells. A specimen was classified as exhibiting PD‐L1 expression if the CPS was ≥ 1 [6, 7, 28].

### 2.4 MSI Status Determination

The microsatellite instability (MSI) status of tumor tissue was evaluated using two methodologies. The first method involved IHC testing to assess the expression of four DNA mismatch repair proteins: MLH1, MSH2, MSH6, and PMS2. Tumors were classified as MSI‐H if there was an absence of expression of more than one of these proteins [29]. The second method utilized next‐generation sequencing, targeting all exons of 706 genes and introns of 39 genes, with an average coverage of at least 1000×. The determination of MSI was performed using bioinformatics approaches. A tumor was classified as MSI‐H if more than 15% of selected microsatellite loci exhibited instability compared to corresponding peripheral blood samples [30].

### 2.5 Blood Collection and Plasma Isolation

Approximately 8 mL of peripheral blood was collected from all participants (*N* = 91) at baseline, and post‐treatment samples were collected after the completion of the standard second 21‐day treatment cycle (−7 to 0 days before the third 21‐day treatment cycle) (*N* = 49). The samples were stored vertically in 10 mL BD Vacutainer K2 EDTA Blood Collection Tubes (BD Biosciences, USA) and were temporarily maintained at 4°C. Within 2 h of collection, plasma was extracted through centrifugation at 3000 rpm for 10 min. Following this, cellular debris was removed by a second centrifugation at 16,000 *g* for 10 min at 4°C [31].

### 2.6 Plasma RNA Extraction

Total RNA was extracted from 300 μL of plasma using the RNAsimple Total RNA Kit (TIANGEN, China), following the manufacturer's instructions. The isolated RNA samples were subsequently stored at −80°C for future use.

### 2.7 sRNA Library Construction and Sequencing

All sRNA library construction and deep sequencing were conducted by BGI (BGI, China). Sequencing libraries were size‐selected for the specific RNA biotypes to be sequenced using an automated gel cutter. The quality and quantity of the libraries were assessed using the Agilent BioAnalyzer 2100. sRNA libraries were constructed following the protocols outlined for the Agilent BioAnalyzer 2100.

### 2.8 Data Processing and Analysis

Mature miRNA sequences, mature tRNA sequences, and rRNA sequences were obtained from miRBase v21 [32], GtRNAdb 2.0 [33], and the National Center for Biotechnology Information (NCBI), respectively. The clean reads were aligned to these reference sequences for annotation using Bowtie [34]. For miRNA annotation, only candidates with one mismatch and no more than two shifts were considered valid matches. The SPORTS 1.1 [35] tool, based on Bowtie, was utilized for the annotation of tRNA and rRNA sequences (Figure 1B).

The ‘filterByExpr’ function from the ‘edgeR’ package was employed with default parameters to retain sRNAs. Specifically, two critical parameters were employed: a minimum count threshold of 10 and a minimum sample requirement, defined as the number of samples with fewer individuals in the group. For example, in the discovery cohort, the ratio of responders to non‐responders is 34:15. Therefore, the application of ‘filterByExpr’ resulted in the retention of sRNAs with at least 10 counts in a minimum of 15 samples. This step effectively removed low‐abundance sRNAs that are unlikely to possess biological significance. Normalization was conducted using the trimmed mean of *M*‐values (TMM) method to account for compositional differences among samples [36]. For the baseline differential analysis, two covariates were incorporated into a linear model framework utilizing the ‘limma’ package. Batch effects were modeled as a categorical variable, and the use of the VEGFR‐2 inhibitor (binary: present/absent) was included to control for its potential confounding effects on immune response. Following the residualization of the normalized counts using the aforementioned model, the Wilcoxon rank‐sum test was applied. A *p*‐value cutoff was then established based on a false discovery rate (FDR) threshold using the Benjamini–Hochberg method [37]. Significance was defined as uncorrected *p* < 0.05 for broad pattern identification, with a fold‐change threshold set at > 1.5.

To identify sRNAs that exhibit significant changes before and after treatment, we performed a Wilcoxon test on the discovery cohort (*N* = 49) to compare sRNA expression levels between pre‐treatment and post‐treatment samples. sRNAs with an uncorrected *p*‐value < 0.05 were retained as candidate treatment‐associated sRNAs. For subgroup analysis of responders (R) and non‐responders (NR), we further stratified the discovery cohort to ensure that observed changes were not confounded by treatment response status. Within each subgroup, we re‐executed the differential expression analysis on the candidate sRNAs identified in Step 1, applying a threshold of *p* < 0.05. For integration and specificity filtering, we merged sRNAs exhibiting significant pre/post‐treatment differences in either the R or NR subgroups. To exclude sRNAs with non‐specific treatment effects, we removed those showing concordant directionality in both subgroups. The final list comprised sRNAs with divergent dynamics or changes exclusive to one subgroup.

### 2.9 Machine Learning Model

The R packages caret [38] and glmnet [39] were employed for data preprocessing and feature selection. Initially, significant differentially expressed sRNA features were pre‐screened within the training set. These features were subsequently filtered using Least Absolute Shrinkage and Selection Operator (LASSO) with leave‐one‐out cross‐validation (LOOCV) across subjects to minimize the risk of overfitting. The baseline prediction model utilized the non‐zero features retained after feature selection to construct three distinct models: Generalized Linear Model (GLM), Random Forest (RF), and Stochastic Gradient Boosting (GBM). The performance of these models was evaluated in both the training and validation cohorts using receiver operating characteristic (ROC) curves [40].

The decision to emphasize the results of the GLM was informed by three key considerations, supported by the performance of the validation cohort: (1) The GLM demonstrated superior generalizability in the independent validation cohort compared to RF and GBM, particularly in maintaining balanced specificity and sensitivity (0.79 and 0.83, respectively), which are critical for clinical implementation, regardless of whether based on the characteristics of the two sRNAs or the combined PD‐L1 CPS characteristics. (2) The perfect specificity (1.00 and 1.00, respectively), sensitivity (1.00 and 1.00, respectively), and Area Under the Curve (AUC) (1.00 and 1.00, respectively) of RF in the discovery cohort, contrasted with its significant performance drop in validation (specificity↓: 32% and 29%, respectively; sensitivity↓: 21% and 23%, respectively; AUC↓: 27% and 28%, respectively), suggesting potential overfitting. GBM exhibited similar validation instability. (3) The linear formulation of GLM allows for explicit coefficient estimates (β_hsa‐miR‐181d‐5p_: 0.002 and 0.002, respectively; β_hsa‐miR‐3916_: −0.004 and −0.003, respectively; β_CPS_: −0.203), enabling clinicians to quantitatively assess biomarker contributions and make risk‐stratified clinical decisions. In contrast, the black‐box nature of RF and GBM renders them less suitable for biomarker studies that require transparent decision pathways.

Furthermore, data from the three sRNAs, PD‐L1 CPS, and serological tumor biomarkers (PLR, NLR, SII, CEA, CA72‐4, CA125, CA19‐9, and AFP) were combined, and features were selected using LASSO with 10‐fold cross‐validation. Subsequently, the GLM model was fitted using the identified non‐zero features (has‐miR‐181d‐5p, has‐miR‐3916, PD‐L1 CPS, CA125, CA19‐9, and AFP).

Additionally, the dynamic monitoring model was assessed separately on the training set, utilizing the non‐zero features retained following feature selection.

### 2.10 Outcomes

The primary endpoint was the overall response rate (ORR), defined as the proportion of patients achieving confirmed CR and PR as assessed by RECIST 1.1 criteria. The secondary endpoints included disease control rate (DCR), PFS, and overall survival (OS). PFS was defined as the time from the start of treatment until the date of disease progression or death resulting from any cause. OS was measured from the start of treatment to the date of death from any cause. DCR was the proportion of patients reaching CR, PR, and SD assessed by RECIST 1.1 criteria.

### 2.11 Sample Size and Statistical Analysis

We established a target AUC of 0.75, treating an AUC of 0.50 as the null hypothesis. We assumed that the proportion of non‐responders to responders was 50%. The statistical power was set at 80%, with a significance level (*α*) of 0.05. Based on these parameters, the required sample size for the discovery cohort was determined to be 44 patients, comprising 29 responders and 15 non‐responders.

Both the ORR and DCR, along with their 95% CIs, were calculated using the ‘binom.test’. A subgroup analysis of ORR was conducted based on baseline patient characteristics. Survival analysis was performed utilizing the R packages ‘survival’ and ‘survminer’. PFS and OS curves were generated using the Kaplan–Meier method, with survival probability plotted against survival time (in months). The significance of survival results was assessed using the log‐rank test, with a *p*‐value of less than 0.05 considered statistically significant.

The Fisher's exact test was employed to analyze and compare categorical variables. The unpaired Wilcoxon test was used for comparisons of independent continuous variables, while the Wilcoxon paired test was applied for paired samples. Additionally, heatmap and volcano analyses of differentially expressed sRNA were conducted using the R packages ‘pheatmap’ and ‘ggplot2’. All statistical analyses were executed using R version 4.2.3.

## 3 Results

### 3.1 Characteristics of Study Subjects

Ninety‐one aGC patients were enrolled in this study between December 2020 and October 2023, including 49 in the discovery cohort and 42 in the validation cohort (Figure 1). Baseline and post‐treatment plasma samples were collected from all participants. The median age of the patients was 63 years (interquartile range: 55–69 years), with the majority being men (82.4%). All patients presented with metastases, including 55 patients (60.5%) with lymph node metastases and 26 patients (28.6%) with liver metastases. Fifty patients (54.9%) exhibited metastases at more than two sites. Additionally, 44 patients (48.4%) had a PD‐L1 CPS ≥ 1, while only three patients (3.3%) were confirmed to be MSI‐H. Eighty‐two patients (90.1%) received combination immunotherapy as first‐line treatment, while 47 patients (51.6%) received a combination of immunotherapy and VEGFR‐2 inhibitors (Table 1).

**TABLE 1:** General characteristics and clinical features for advanced gastric cancer patients included in this study.

	Discovery cohort (*N* = 49)			Validation cohort (*N* = 42)			
Variables	Non‐responder (*N* = 15)	Responder (*N* = 34)	*p*	Non‐responder (*N* = 14)	Responder (*N* = 28)	*p*	Total (*N* = 91)
Age, years							
Median (IQR)	65.0 (49.0, 67.5)	61.0 (54.5, 66.0)	0.750	65.5 (55.2, 71.0)	67.5 (56.5, 70.0)	0.940	63.0 (55.0, 69.0)
Sex, *n* (%)							
Male	13 (86.7)	30 (88.2)		10 (71.4)	22 (78.6)	0.900	75 (82.4)
Female	2 (13.3)	4 (11.8)	1.000	4 (28.6)	6 (21.4)		16 (17.6)
Histological grade, *n* (%)							
Poorly differentiated	8 (53.3)	12 (35.3)	0.512	5 (35.7)	14 (50.0)	0.537	39 (42.9)
Moderately to poorly differentiated	3 (20.0)	12 (35.3)		3 (21.4)	6 (21.4)		24 (26.4)
Moderately differentiated	4 (26.7)	5 (14.7)		5 (35.7)	8 (28.6)		22 (24.2)
Well to moderately differentiated	0 (0.0)	1 (2.9)		0 (0.0)	0 (0.0)		1 (1.1)
Well differentiated	0 (0.0)	0 (0.0)		1 (7.1)	0 (0.0)		1 (1.1)
Unknown	0 (0.0)	4 (11.8)		0 (0.0)	0 (0.0)		4 (4.4)
Lauren Classification, *n* (%)							
Diffuse	4 (26.7)	9 (26.5)	0.406	1 (7.1)	7 (25.0)	0.179	21 (23.1)
Intestinal	8 (53.3)	10 (29.4)		9 (64.3)	11 (39.3)		38 (41.8)
Mixed	2 (13.3)	8 (23.5)		3 (21.4)	10 (35.7)		23 (25.3)
Unknown	1 (6.7)	7 (20.6)		1 (7.1)	0 (0.0)		9 (9.9)
Primary tumor site, *n* (%)							
Antrum	4 (26.7)	8 (23.5)	1.000	3 (21.4)	13 (46.4)	0.120	28 (30.8)
Body	6 (40.0)	15 (44.1)		5 (35.7)	6 (21.4)		32 (35.2)
Cardia	5 (33.3)	10 (29.4)		4 (28.6)	9 (32.1)		28 (30.8)
Fundus	0 (0.0)	1 (2.9)		2 (14.3)	0 (0.0)		3 (3.3)
ECOG performance status^a^, *n* (%)							
0	2 (13.3)	10 (29.4)	0.400	3 (21.4)	8 (28.6)	0.900	23 (25.3)
1	13 (86.7)	24 (70.6)		11 (78.6)	20 (71.4)		68 (74.7)
Metastasis site, *n* (%)							
Liver and lymph node	4 (26.7)	8 (23.5)	0.990	4 (28.6)	5 (17.9)	0.850	21 (23.1)
Liver only	4 (26.7)	10 (29.4)		4 (28.6)	8 (28.6)		26 (28.6)
Lymph node only	6 (40.0)	13 (38.2)		4 (28.6)	11 (39.3)		34 (37.4)
Other	1 (6.7)	3 (8.8)		2 (14.3)	4 (14.3)		10 (11.0)
Number of target lesions, *n* (%)							
1	8 (53.3)	19 (55.9)	1.000	3 (21.4)	11 (39.3)	0.420	41 (45.1)
2 or more	7 (46.7)	15 (44.1)		11 (78.6)	17 (60.7)		50 (54.9)
Lines of therapy, *n* (%)							
1 L	11 (73.3)	29 (85.3)	0.550	14 (100.0)	28 (100.0)	NA	82 (90.1)
2 L	4 (26.7)	5 (14.7)		0 (0.0)	0 (0.0)		9 (9.9)
PD‐L1 status^b^, *n* (%)							
Negative	9 (60.0)	10 (29.4)	0.194	8 (57.1)	10 (35.7)	0.418	37 (40.7)
Positive	6 (40.0)	19 (55.9)		5 (35.7)	14 (50.0)		44 (48.4)
Unknown	0 (0.0)	5 (14.7)		1 (7.1)	4 (14.3)		10 (11.0)
MSI status^c^, *n* (%)							
MSI‐H	0 (0.0)	2 (5.9)	0.398	0 (0.0)	1 (3.6)	1.000	3 (3.3)
MSI‐L	1 (6.7)	0 (0.0)		0 (0.0)	0 (0.0)		1 (1.1)
MSS	14 (93.3)	29 (85.3)		11 (78.6)	24 (85.7)		78 (85.7)
Unknown	0 (0.0)	3 (8.8)		3 (21.4)	3 (10.7)		9 (9.9)
HER2 status, *n* (%)							
Negative	10 (66.7)	22 (64.7)	1.000	8 (57.1)	12 (42.9)	NA	52 (57.1)
Positive	3 (20.0)	6 (17.6)		0 (0.0)	0 (0.0)		9 (9.9)
Unknown	2 (13.3)	6 (17.6)		6 (42.9)	16 (57.1)		30 (33.0)
Immunotherapy treatment (%)							
Combination chemotherapy	12 (80.0)	17 (50.0)	0.100	8 (57.1)	7 (25.0)	0.090	44 (48.4)
Combination chemotherapy and VEGFR‐2 inhibitors	3 (20.0)	17 (50.0)		6 (42.9)	21 (75.0)		47 (51.6)
Best overall response to combination immunotherapy (%)							
Partial response	0 (0.0)	29 (85.3)	< 0.001	0 (0.0)	23 (82.1)	< 0.001	52 (57.1)
Stable disease	11 (73.3)	5 (14.7)		9 (64.3)	5 (17.9)		30 (33.0)
Progressive disease	4 (26.7)	0 (0.0)		5 (35.7)	0 (0.0)		9 (9.9)

Abbreviations: MSI, microsatellite instability; MSS, microsatellite stable.

^a^ ECOG performance status, Eastern Cooperative Oncology Group performance status.

^b^ Negative, combined positive score < 1; Positive, combined positive score ≥ 1.

^c^ MSI, microsatellite instability; MSS, microsatellite stable.

### 3.2 Response to Combination Immunotherapy

**FIGURE 2:**
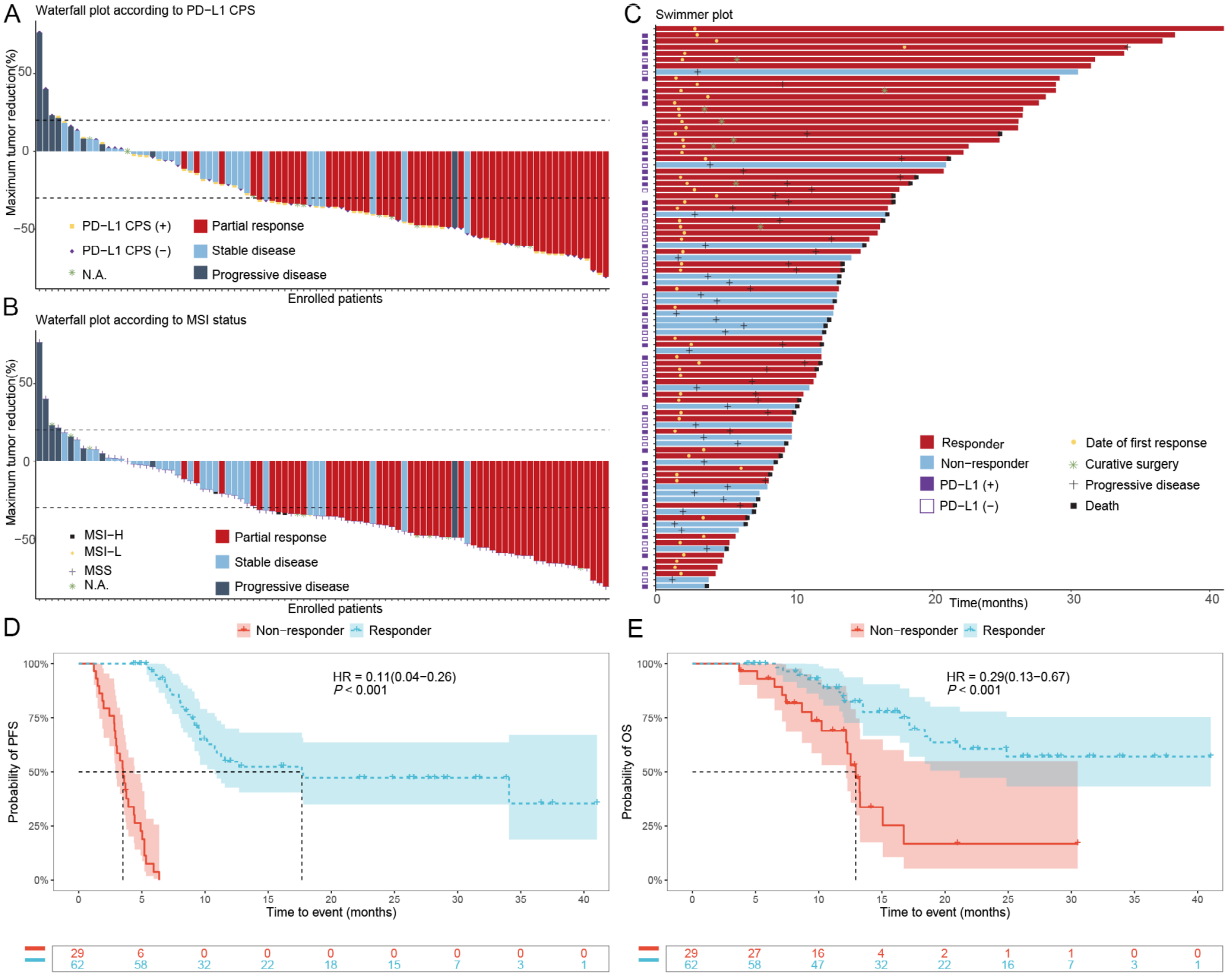
Response to combination immunotherapy in patients with advanced gastric cancer. (A) Waterfall plot depicting the response to combination immunotherapy based on PD‐L1 combined positive score (CPS). The Y‐axis represents the percentage of maximum tumor reduction, assessed according to RECIST 1.1 criteria. The lower dotted line represents a tumor reduction of 30% per RECIST 1.1 (partial response, PR), while the upper dashed line indicates a tumor increase of 20% (progressive disease, PD). (B), Waterfall plot stratified by microsatellite instability (MSI) status, with the Y‐axis again reflecting the percentage of maximum tumor reduction as per RECIST 1.1 criteria. (C) Swimmer plot detailing individual patient data; each lane represents one patient. (D) Kaplan–Meier (KM) curve analysis comparing progression‐free survival (PFS) between responders and non‐responders. (E) KM curve analysis comparing overall survival (OS) between responders and non‐responders. The *p*‐value was calculated using the log‐rank test. Horizontal and vertical dashed lines indicate the median survival time, and the shaded ribbon represents the confidence interval. The X‐axis denotes the duration of immunotherapy for each patient. Patient identity numbers are provided in Table S2. NE, not estimable; NA, not available.

During a median follow‐up period of 12.9 months, 34 deaths occurred among 91 patients. Response evaluations revealed PR in 52 (57.1%) patients, SD in 31 (34.1%) patients, and PD in 8 patients (Table 2). Consequently, the ORR was 57.1% (95% CI: 46.3%–67.5%) and the DCR was 91.2% (95% CI: 83.4%–96.1%). Among the 52 patients with PR, 40 experienced a reduction in tumor burden exceeding 50% (Figure 2A).

**TABLE 2:** Tumor response assessed by Response Evaluation Criteria in Solid Tumors (version 1.1).

Response	Total (*N* = 91)	PD‐L1 CPS < 1 (*N* = 37)	PD‐L1 CPS ≥ 1 (*N* = 44)
Partial response, *n* (%)	52 (57.1)	16 (43.2)	28 (63.6)
Stable disease, *n* (%)	31 (34.1)	16 (43.2)	13 (29.5)
Progressive disease, *n* (%)	8 (8.8)	5 (13.5)	3 (6.8)
Objective response rate, % (95% CI)	57.1 (46.3–67.5)	43.2 (27.1–60.5)	63.6 (47.8–77.6)
Disease control rate, % (95% CI)	91.2 (83.4–96.1)	86.5 (71.2–95.5)	93.2 (81.3–98.6)

Abbreviation: CPS, combined positive score.

We initially analyzed responses based on PD‐L1 CPS positivity (Figure 2A). Among the 81 patients with available PD‐L1 CPS data, the ORRs for PD‐L1(+) and PD‐L1(−) were 63.6% (95% CI: 47.8%–77.6%) and 43.2% (95% CI: 27.1%–60.5%), respectively (*p* = 0.107; Figure 2A, Table 2). A higher ORR (68.1%, 95% CI: 52.9%–80.9%) was observed in patients treated with a combination of immunotherapy with chemotherapy and VEGFR‐2 inhibitors (Table S1). Notably, all three patients with MSI‐H exhibited durable responses lasting over 28 months and remained in remission at the time of analysis, despite one patient not achieving PR (Figure 2B,C).

The median PFS of responders was 17.7 months (95% CI: 10.8–NE), whereas the median OS was not yet mature (95% CI: 21.2–NE). In contrast, the mPFS of non‐responders was 3.5 months (95% CI: 3.0–4.4), with a mOS of 12.9 months (95% CI: 12.2–NE). Kaplan–Meier (KM) plot analysis demonstrated that responders had significantly better PFS and OS compared to non‐responders (*p* < 0.001; Figure 2D,E).

We further analyzed the relationship between clinical features and response to combination immunotherapy (Figure S1). Notably, we observed a significant difference in baseline CEA levels between responders and non‐responders (*p* = 0.031). However, no significant differences were detected in inflammation scores, including the PLR, NLR, and SII, as well as tumor biomarkers such as CA72‐4, CA125, CA19‐9, and AFP. The AUCs for serological inflammation biomarkers ranged from 0.53 to 0.60 for PLR, NLR, and SII, while the AUCs for tumor biomarkers ranged from 0.52 to 0.64 for CEA, CA72‐4, CA125, CA19‐9, and AFP (Figure S1, Table S3).

### 3.3 sRNA Signatures in Baseline Plasma of aGC Patients

**FIGURE 3:**
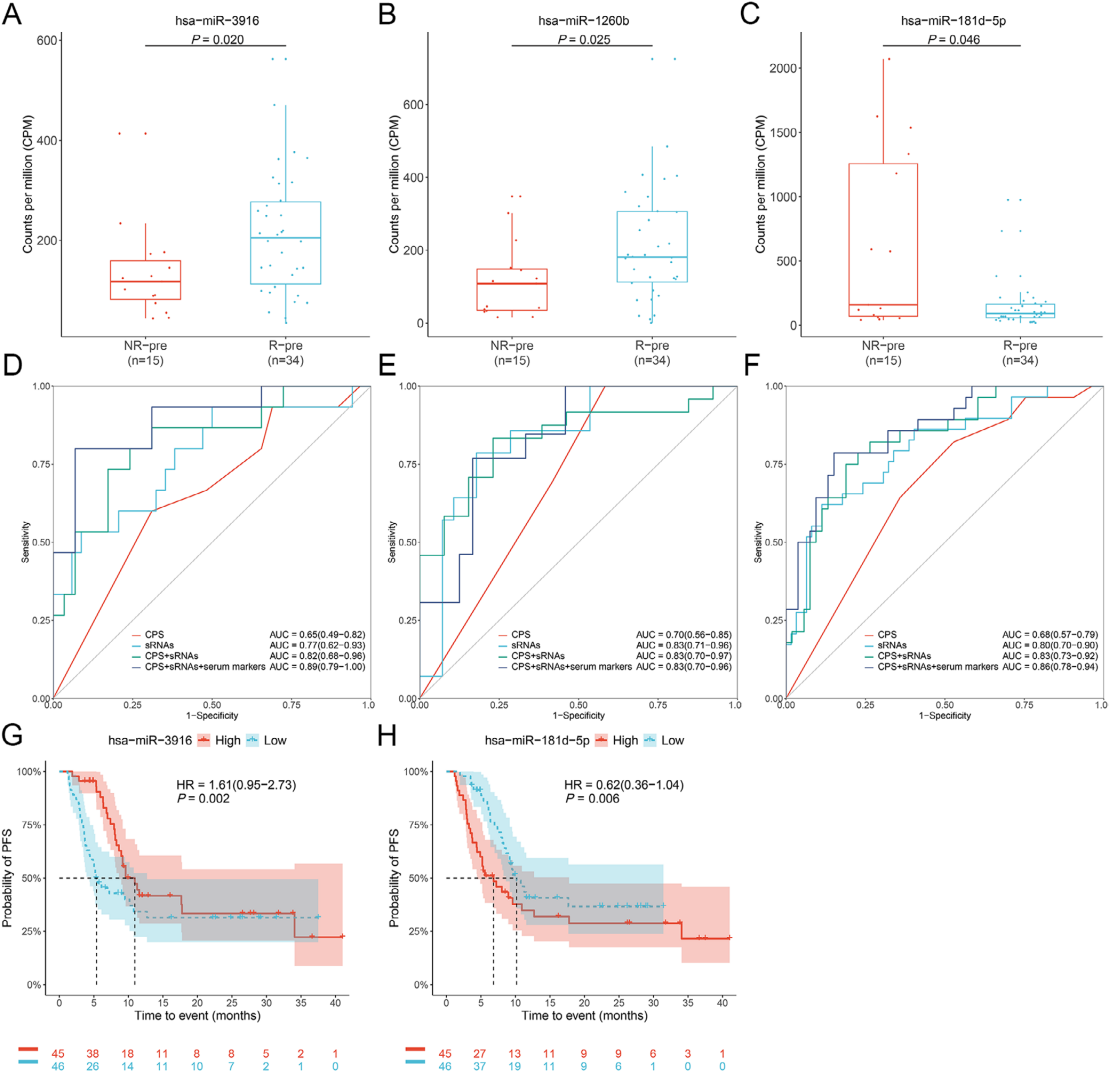
Differential baseline plasma small RNA (sRNA) analysis between responders and non‐responders in patients with advanced gastric cancer (aGC). (A–C) Box plots showing the counts per million (CPM) of hsa‐miR‐3916, hsa‐miR‐181d‐5p, and has‐miR‐1260b between responders and non‐responders in the discovery cohort. The *p*‐value was calculated using Wilcoxon rank sum tests. R‐pre, responder pre‐treatment; NR‐pre, non‐responder pre‐treatment. Receiver operating characteristic (ROC) curves illustrate the performance of the baseline prediction model classification in the discovery cohort (D), validation cohort (E), and combined datasets (F). These curves are based on PD‐L1 combined positive score (CPS), two sRNAs, the integration of PD‐L1 CPS with two sRNAs, and the combination of PD‐L1 CPS and serological tumor biomarkers (CA125, CA19‐9, AFP) with two sRNAs. The shaded ribbon represents the confidence interval. For each sRNA analyzed, patients with aGC were categorized into ‘high (H)’ or ‘low (L)’ groups based on the expression levels of the sRNA. Kaplan–Meier (KM) curve analysis of (G) hsa‐miR‐3916, and (H) hsa‐miR‐181d‐5p with PFS of patients with aGC. The *p*‐value was calculated using the log‐rank test. Horizontal and vertical dashed lines indicate the median survival time, and the shaded ribbon represents the confidence interval.

To investigate the potential of sRNAs as novel biomarkers for combination immunotherapy in aGC, we conducted preliminary screenings using small RNA sequencing on baseline plasma samples from both discovery and validation cohorts (Figure 1). We found that, as compared to non‐responders, levels of hsa‐miR‐3916 and hsa‐miR‐1260b were significantly higher, while hsa‐miR‐181d‐5p level was significantly lower in responders in the discovery cohort (Figure 3A–C, Figure S2A, Table S4).

To differentiate responders from non‐responders based on baseline plasma sRNA features, we developed a prediction model utilizing non‐zero features retained after LASSO feature selection. Specifically, we employed two features, hsa‐miR‐3916 and hsa‐miR‐181d‐5p, to train three distinct machine learning models: GLM, RF, and GBM. The models were trained on data from 34 responders and 15 non‐responders within the discovery cohort, followed by validation using data from 28 responders and 14 non‐responders in the validation cohort. Ultimately, GLM was chosen for its applicability and interpretability. The AUCs were 0.77 (95% CI: 0.62–0.93; Figure 3D, Figure S3A) in the discovery cohort, 0.83 (95% CI: 0.71–0.96; Figure 3E, Figure S3B) in the validation cohort, and 0.80 (95% CI: 0.70–0.90; Figure 3F, Figure S3C) in the combined dataset. Additionally, patients with high levels of hsa‐miR‐3916 (median: 10.93 vs. 5.39 months, *p* = 0.002, Figure 3G) or low levels of hsa‐miR‐181d‐5p showed significantly improved PFS (median: 10.17 vs. 6.83 months, *p* = 0.006, Figure 3H). However, these levels were not associated with OS in aGC patients (Figure S2C,D).

Furthermore, we incorporated PD‐L1 CPS positivity alongside these two features, hsa‐miR‐3916 and hsa‐miR‐181d‐5p, to refine the prediction model. The AUCs were 0.82 (95% CI: 0.68–0.96; Figure 3D, Figure S3D) in the discovery cohort, 0.83 (95% CI: 0.70–0.97; Figure 3E, Figure S3E) in the validation cohort, and 0.83 (95% CI: 0.73–0.92; Figure 3F, Figure S3F) in the combined dataset. These results suggest the potential of the model based on baseline plasma sRNA features, combined with PD‐L1 CPS positivity, to predict responses to combination immunotherapy in patients with aGC.

Additionally, when combining PD‐L1 CPS and serological tumor biomarkers with these two sRNAs, the AUCs were 0.89 (95% CI; 0.79–1.00; Figure 3D) in the discovery cohort, 0.83 (95% CI; 0.70–0.96; Figure 3E) in the validation cohort, and 0.86 (95% CI: 0.78–0.94; Figure 3F) in the combined dataset. These results suggest the potential of the model based on baseline plasma sRNA features, combined with PD‐L1 CPS positivity, to predict responses to combination immunotherapy in patients with aGC.

### 3.4 sRNA Signature Changes in Paired Plasma of aGC Patients Induced by Combination Immunotherapy

**FIGURE 4:**
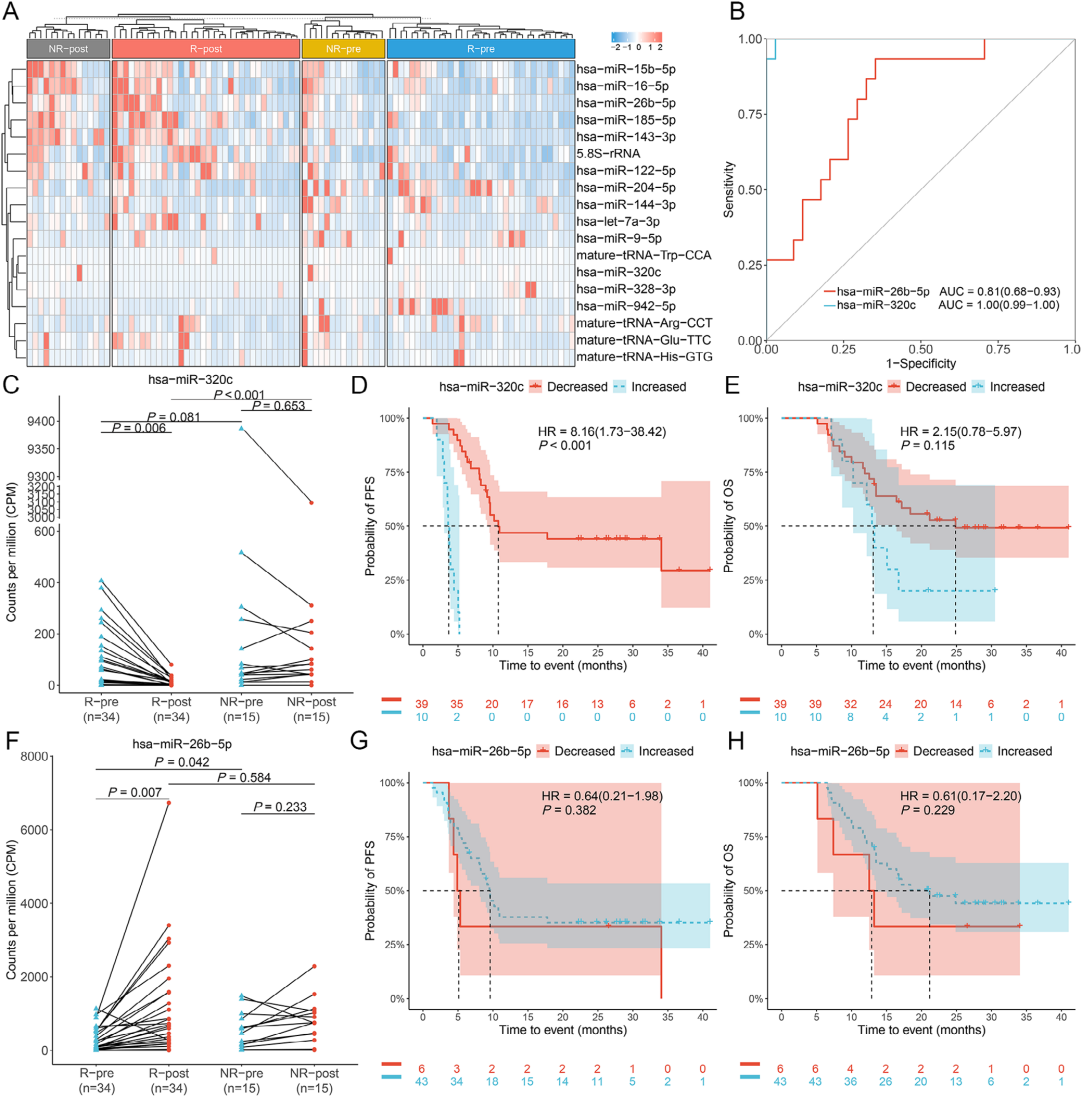
Plasma small RNA (sRNA) levels pre‐ and post‐treatment in responders (R) and non‐responders (NR) following combination immunotherapy. (A) Differential plasma sRNA levels between pre‐ and post‐treatment in responders and non‐responders. (B) Receiver operating characteristic (ROC) curves classifying responders and non‐responders based on post‐treatment dynamic changes in the two individual sRNA features identified through Least Absolute Shrinkage and Selection Operator (LASSO) feature selection. Counts per million (CPM) of hsa‐miR‐320C (C) and hsa‐miR‐26b‐5p (F) compared across R‐pre, R‐post, NR‐pre, and NR‐post in the discovery cohort. *p*‐value was calculated using Wilcoxon rank sum tests. R‐pre, responder pre‐treatment; R‐post, responder post‐treatment; NR‐pre, non‐responder pre‐treatment; NR‐post, non‐responder post‐treatment. (D, E) Patients stratified by changes in plasma levels of hsa‐miR‐320c following combination immunotherapy relative to baseline. KM curve analysis comparing progression‐free survival (PFS, D) and overall survival (OS, E) between responders and non‐responders. (G, H) Patients stratified by changes in plasma levels of hsa‐miR‐26b‐5p following combination immunotherapy relative to baseline. KM curve analysis comparing PFS (G) and OS (H) between responders and non‐responders. *p*‐values were calculated using the log‐rank test. Horizontal and vertical dashed lines indicate median survival time; shaded ribbons represent confidence intervals.

We compared the sRNA profiles before and after therapy in the discovery cohort and identified 18 sRNAs with significant changes in plasma levels attributable to combination immunotherapy (Table S5). In responders, combination immunotherapy significantly upregulated six sRNAs and downregulated another six; conversely, only one sRNA was upregulated in nonresponders (Figure 4A, Table S6).

To differentiate responders from non‐responders based on paired plasma sRNA features, we further screened for differentially expressed sRNAs using LASSO feature selection and identified dynamic changes in hsa‐miR‐320c and hsa‐miR‐26b‐5p plasma levels for monitoring the efficacy of combination immunotherapy, with AUCs of 1.00 (95% CI: 0.73–0.92) and 0.81 (95% CI: 0.68–0.93) (Figure 4B), respectively. Responders demonstrated a significant downregulation of hsa‐miR‐320c (*p* = 0.006) (Figure 4C, Table S6) and a significant upregulation of hsa‐miR‐26b‐5p (*p* = 0.007) (Figure 4F, Table S6) following combination immunotherapy.

Furthermore, patients with decreased levels of hsa‐miR‐320c demonstrated a trend towards improved PFS (median: 9.17 vs. 3.03 months, *p* < 0.001) (Figure 4D) and OS (median: 16.43 vs. 10.23 months, *p* = 0.115) (Figure 4E). In contrast, patients with elevated levels of hsa‐miR‐26b‐5p also demonstrated a trend towards improved PFS (median: 9.17 vs. 3.03 months, *p* = 0.382) (Figure 4G) and OS (median: 21.17 vs. 12.88 months, *p* = 0.229) (Figure 4H), although these results did not reach statistical significance. These findings indicate the potential of dynamic changes in hsa‐miR‐320c and hsa‐miR‐26b‐5p plasma levels as monitoring biomarkers for response to combination immunotherapy.

## 4 Discussion

Among the 91 aGC patients of this study, the ORRs were 43.2% for those with PD‐L1 CPS < 1 and 63.6% for CPS ≥ 1, indicating a positive correlation between PD‐L1 expression levels and response to combination immunotherapy. These real‐world results are comparable to those reported in the KEYNOTE‐059 trial [41]: 37.5% and 68.8% for PD‐L1 CPS < 1 and CPS ≥ 1, respectively. These findings highlight the varying ORRs among patients treated with immunotherapy and suggest the validity of using PD‐L1 CPS as a predictive biomarker for immunotherapy outcomes. Furthermore, data from the KEYNOTE‐059, KEYNOTE‐061, and KEYNOTE‐062 studies showed ORRs of 57%, 47%, and 57% for patients with MSI‐H aGC, respectively [42], demonstrating the potential of MSI‐H status as another important biomarker for predicting treatment responsiveness. However, it's noteworthy that only 3 out of the 91 patients were identified as MSI‐H, with two of them showing an overall response, suggesting that while MSI‐H can be indicative of a favorable response, its prevalence in this cohort was low. Additionally, the investigation into other inflammation scores (like PLR, NLR, and SII) and conventional serological tumor biomarkers (including CEA, CA72‐4, CA125, CA19‐9, and AFP) revealed limited effectiveness in distinguishing responders from non‐responders to combination immunotherapy. In summary, while PD‐L1 CPS remains a key biomarker for assessing immunotherapy efficacy, there is a clear need for the identification of additional biomarkers that can better predict patient responses to immunotherapy in aGC.

Importantly, we identified two sRNAs, hsa‐miR‐3916 and hsa‐miR‐181d‐5p, as potential predictors for distinguishing responders from non‐responders based on baseline plasma. These sRNAs were utilized to develop a predictive model, demonstrating superior performance in terms of AUC compared to serological inflammation biomarkers and relevant tumor biomarkers. Notably, when combined with PD‐L1 CPS positivity, the AUC for the prediction model reached 0.83 in the validation cohort. This combination has the potential to enhance patient stratification, identifying those more likely to benefit from combination immunotherapy. The stability and reproducibility of plasma sRNA levels are crucial for their clinical application. Unlike tissue samples, which can vary due to factors such as tumor heterogeneity, the identified plasma sRNAs may serve as more consistent and accessible biomarkers.

The miRNA‐181 family exhibits dual regulatory roles in oncogenesis, functioning as both tumor‐promoting and tumor‐suppressing microRNAs through the modulation of multiple signaling pathways, particularly the PI3K/AKT, MAPK, TGF‐β, Wnt, NF‐κB, and Notch cascades [43]. Emerging evidence indicates that the overexpression of exosomal hsa‐miR‐181d‐5p confers resistance to 5‐fluorouracil by directly targeting neurocalcin δ (NCALD) [44]. Interestingly, hsa‐miR‐181d‐5p participates in a genome instability‐associated competing endogenous RNA (ceRNA) network comprising four distinct regulatory axes which has been identified as a crucial modulator of the tumor microenvironment, cancer stemness and resistance to immunotherapy [45]. In contrast to the well‐characterized miR‐181 family, the biological functions of hsa‐miR‐3916 remain poorly understood. Mechanistic studies demonstrate that the overexpression of hsa‐miR‐3916 significantly downregulates the protein expression of pyruvate dehydrogenase kinase 1 (PDK1), thereby attenuating glycolytic metabolism and suppressing the migratory and invasive capacities of prostate cancer (PCa) cells. These findings collectively suggest that hsa‐miR‐3916 may serve as a novel tumor suppressor in the pathogenesis of PCa [46]. Although the current study did not directly investigate the functional crosstalk between hsa‐miR‐3916 and hsa‐miR‐181d‐5p, their complementary targeting of metabolic reprogramming (PDK1), drug resistance (NCALD), and TME modulation pathways suggests potential synergistic mechanisms in shaping immunotherapy responsiveness. Future studies employing dual miRNA inhibition/overexpression models in patient‐derived organoids will be crucial to dissect their interplay.

Furthermore, we identified two additional sRNAs, hsa‐miR‐320c and hsa‐miR‐26b‐5p, that may serve as biomarkers for monitoring the efficacy of combination immunotherapy. Notably, elevated levels of hsa‐miR‐320 have been associated with poor prognosis and increased risk of metastasis in ovarian cancer [47]. Overexpression of hsa‐miR‐320 promotes B‐cell proliferation by suppressing the expression of Phosphatase and Tensin Homolog (PTEN) and enhancing cyclin D1 expression [48]. Additionally, high expression of hsa‐miR‐320c has been correlated with poor prognosis and reduced response to anti‐PD‐1 treatment in patients with NSCLC [25]. Conversely, hsa‐miR‐26b‐5p is frequently downregulated in various cancers and has been shown to inhibit tumor growth and proliferation, and induce apoptosis [49, 50]. Low expression levels of miR‐26b have been correlated with poor prognosis in colorectal cancer (CRC) and NSCLC [51]. We found that among aGC patients following combination immunotherapy, responders exhibited significant downregulation of hsa‐miR‐320c and upregulation of hsa‐miR‐26b‐5p compared to nonresponders. This expression pattern was associated with improved PFS and OS, indicating a favorable outcome for patients with aGC. These findings suggest that decreased plasma levels of hsa‐miR‐320c and increased levels of hsa‐miR‐26b‐5p may represent a positive response in aGC patients. However, plasma samples from validation cohorts post anti‐PD‐1 treatment were not available for analysis in this study; thus, future investigations are warranted for further exploration and validation. Nevertheless, our findings suggest that the ability to use a simple blood sample to assess sRNA levels allows for non‐invasive monitoring of tumor dynamics. This is particularly valuable for tracking response to combination immunotherapy and making timely adjustments to therapeutic strategies, thereby enhancing patient management.

There are several limitations in this study. The limited availability of tissue samples for TMB and EBV detection restricted our ability to compare these potential biomarkers with others, which is essential for evaluating their predictive accuracy. Furthermore, the small sample size is a further limitation which may pose inherent challenges for sRNA profiling, including the risk of false‐positive findings and reduced statistical power to detect subtle effects. Although we employed a discovery/validation cohort approach to mitigate these risks, the limited cohort size may still constrain the generalizability and robustness of our findings. Moreover, due to the challenges associated with paired longitudinal sample collection, the validation cohort did not verify the results of dynamic monitoring of longitudinal samples in the discovery cohort. Therefore, these results should be interpreted with caution and considered as hypothesis‐generating. The two sRNAs employed for predicting combination immunotherapy efficacy, as well as the two sRNAs used for dynamic monitoring of treatment efficacy, require validation in larger cohorts to confirm their potential as biomarkers for combination immunotherapy.

## 5 Conclusion

In conclusion, we identified two plasma‐derived sRNAs before therapy and two additional plasma‐derived sRNAs after therapy as potential biomarkers of combination immunotherapy in patients with aGC. These biomarkers outperformed existing serological indicators, including inflammatory factors, serological tumor biomarkers, and PD‐L1 CPS at the tumor tissue level. They could serve as non‐invasive indicators for longitudinal liquid biopsies to assess patient eligibility for combination immunotherapy or, at the very least, act as complementary testing tools. However, extensive cohort validation and functional studies of these potential biomarkers are essential before their application in clinical practice.

## Author Contributions

**Jingshuai Fang:** methodology, validation, investigation, writing – original draft, visualization, project administration, data curation, software, formal analysis. **Yan Sun:** methodology, project administration, resources, data curation, investigation. **Yuhui Yu:** project administration, supervision, validation, investigation, methodology, data curation. **Zheng Fu:** methodology, supervision, investigation. **Yitong Tian:** investigation, data curation, project administration. **Yizhang Chen:** data curation, project administration, investigation. **Fen Guo:** investigation, project administration, data curation. **Jie Tang:** investigation, data curation, project administration. **Caiwang Yan:** supervision, investigation. **Xi Chen:** methodology, conceptualization, funding acquisition. **Xiaofeng Chen:** conceptualization, funding acquisition, resources, data curation, validation. **Guangfu Jin:** conceptualization, funding acquisition, writing – review and editing, project administration, resources.

## Ethics Statement

This study was approved by the institution's ethical committees of Nanjing Medical University (approval number: NJMUIRB (2021) 589) and The First Affiliated Hospital of Nanjing Medical University (approval numbers: 2020‐SR‐002 and 2020‐SR‐558).

## Consent

All participants provided written informed consent prior to their inclusion in the study.

## Conflicts of Interest

The authors declare no conflicts of interest.

## Supporting information


**Appendix S1:** cam471339‐sup‐0001‐AppendixS1.docx.
**Figure S1:** Inflammation scores and cancer biomarkers in responders and non‐responders among advanced gastric cancer patients. (A–C) Comparison of inflammatory parameters, including platelet‐to‐lymphocyte ratio (PLR) (A); neutrophil‐to‐lymphocyte ratio (NLR) (B); and systemic immune‐inflammation index (SII) (C) between responders and non‐responders. (D–H) Comparison of cancer biomarkers, including carcinoembryonic antigen (CEA) (D); carbohydrate antigen (CA) 72‐4 (E); CA125 (F); CA19‐9 (G); and alpha‐fetoprotein (AFP) (H) between responders and non‐responders. *p*‐values were calculated using Wilcoxon rank sum tests. R‐pre, responder pre‐treatment; NR‐pre, non‐responder pre‐treatment. (I, J) Receiver Operating Characteristic (ROC) curve illustrating the performance of inflammation scores (I) and cancer biomarkers (J) in classifying responders and non‐responders to combination immunotherapy.
**Figure S2:** Clinical outcome prediction by small RNAs in baseline plasma samples of advanced gastric cancer patients. (A) Volcano plot illustrating the baseline plasma sRNAs differentially expressed between responders and non‐responders in the discovery cohort. (B) Spearman rank correlations between all two sRNAs, PD‐L1 CPS, and serological tumor biomarkers (CA125, CA19‐9, AFP) in the discovery cohort. An elliptical shape in the grid indicates a significant correlation, with correlation *p*‐values corrected for multiple testing using the false discovery rate (FDR) approach (two‐sided rank correlation *t*‐test; FDR ≤ 0.05 cutoff for inclusion in the figure). The size and color intensity of the elliptical shapes reflect the magnitude of the correlation, as indicated in the color legend. Blue elliptical shapes represent positive correlations, while red elliptical shapes signify negative correlations. For each small RNA (sRNA) analyzed, patients with advanced gastric cancer (aGC) were categorized into ‘high (H)’ or ‘low (L)’ groups based on the expression levels of the sRNA. The Kaplan–Meier (KM) curve analyses compared overall survival (OS) for (C) hsa‐miR‐3916, and (D) hsa‐miR‐181d‐5p. *p*‐values were calculated using the log‐rank test. Horizontal and vertical dashed lines indicate median survival time; shaded ribbons represent confidence intervals.
**Figure S3:** Classification of responders and non‐responders using RF, GLM and GBM models based on plasma small RNA (sRNA) features in advanced gastric cancer patients. Performance of Random Forest (RF), Generalized Linear Model (GLM), and Stochastic Gradient Boosting (GBM) model using plasma sRNA features to predict responders to combination immunotherapy in the discovery cohort (A), the validation cohort (B), and the combined dataset (C). Performance of RF, GLM, and GBM models using plasma sRNA features combined with PD‐L1 CPS positivity to predict responders to combination immunotherapy in the discovery cohort (D), the validation cohort (E), and the combined dataset (F).
**Table S1:** Tumor response assessed by Response Evaluation Criteria in Solid Tumors (version 1.1).
**Table S2:** Characteristics of combination immunotherapy responses in terms of MSI and PD‐L1 IHC.
**Table S3:** Summary of patients' clinical information in discovery and validation cohorts.
**Table S4:** Differential expression of small RNAs between responders and non‐responders among aGC patients in baseline plasma samples.
**Table S5:** Differential expression of small RNAs between pre‐ and post‐treatment among aGC patients in paired plasma samples.
**Table S6:** Differential plasma small RNA levels between pre‐ and post‐treatment in responders and non‐responders of aGC patients in the discovery cohort.

## Data Availability

The data that support the findings of this study are available on request from the corresponding author. The data are not publicly available due to privacy or ethical restrictions.
