# Supplementary material for: Plasma Small RNAs as Predictive and Monitoring Biomarkers for Combination Immunotherapy in Advanced Gastric Cancer
Source: Cancer Med. 2025 Nov 4;14(21):e71339. doi: 10.1002/cam4.71339 (PMC12585256; doi:10.1002/cam4.71339)
Supplement: Supplementary file 1 — Appendix S1: cam471339‐sup‐0001‐AppendixS1.docx. Figure S1: Inflammation scores and cancer biomarkers in responders and non‐responders among advanced gastric cancer patients. (A–C) Comparison of inflammatory parameters, including platelet‐to‐lymphocyte ratio (PLR) (A); neutrophil‐to‐lymphocyte ratio (NLR) (B); and systemic immune‐inflammation index (SII) (C) between responders and non‐responders. (D–H) Comparison of cancer biomarkers, including carcinoembryonic antigen (CEA) (D); carbohydrate antigen (CA) 72‐4 (E); CA125 (F); CA19‐9 (G); and alpha‐fetoprotein (AFP) (H) between responders and non‐responders. p‐values were calculated using Wilcoxon rank sum tests. R‐pre, responder pre‐treatment; NR‐pre, non‐responder pre‐treatment. (I, J) Receiver Operating Characteristic (ROC) curve illustrating the performance of inflammation scores (I) and cancer biomarkers (J) in classifying responders and non‐responders to combination immunotherapy. Figure S2: Clinical outcome prediction by small RNAs in baseline plasma samples of advanced gastric cancer patients. (A) Volcano plot illustrating the baseline plasma sRNAs differentially expressed between responders and non‐responders in the discovery cohort. (B) Spearman rank correlations between all two sRNAs, PD‐L1 CPS, and serological tumor biomarkers (CA125, CA19‐9, AFP) in the discovery cohort. An elliptical shape in the grid indicates a significant correlation, with correlation p‐values corrected for multiple testing using the false discovery rate (FDR) approach (two‐sided rank correlation t‐test; FDR ≤ 0.05 cutoff for inclusion in the figure). The size and color intensity of the elliptical shapes reflect the magnitude of the correlation, as indicated in the color legend. Blue elliptical shapes represent positive correlations, while red elliptical shapes signify negative correlations. For each small RNA (sRNA) analyzed, patients with advanced gastric cancer (aGC) were categorized into ‘high (H)’ or ‘low (L)’ groups based on th [file CAM4-14-e71339-s001.docx]

**Supplementary Fig. 1: Inflammation scores and cancer biomarkers in responders and non-responders among advanced gastric cancer patients.** (**A-C**) Comparison of inflammatory parameters, including platelet-to-lymphocyte ratio (PLR) (**A**); neutrophil-to-lymphocyte ratio (NLR) (**B**); and systemic immune-inflammation index (SII) (**C**) between responders and non-responders. (**D-H**) Comparison of cancer biomarkers, including carcinoembryonic antigen (CEA) (**D**); carbohydrate antigen (CA) 72-4 (**E**); CA125 (**F**); CA19-9 (**G**); and alpha-fetoprotein (AFP) (**H**) between responders and non-responders. P-values were calculated using Wilcoxon rank sum tests. R-pre, responder pre-treatment; NR-pre, non-responder pre-treatment. (**I-J**) Receiver Operating Characteristic (ROC) curve illustrating the performance of inflammation scores (**I**) and cancer biomarkers (**J**) in classifying responders and non-responders to combination immunotherapy.


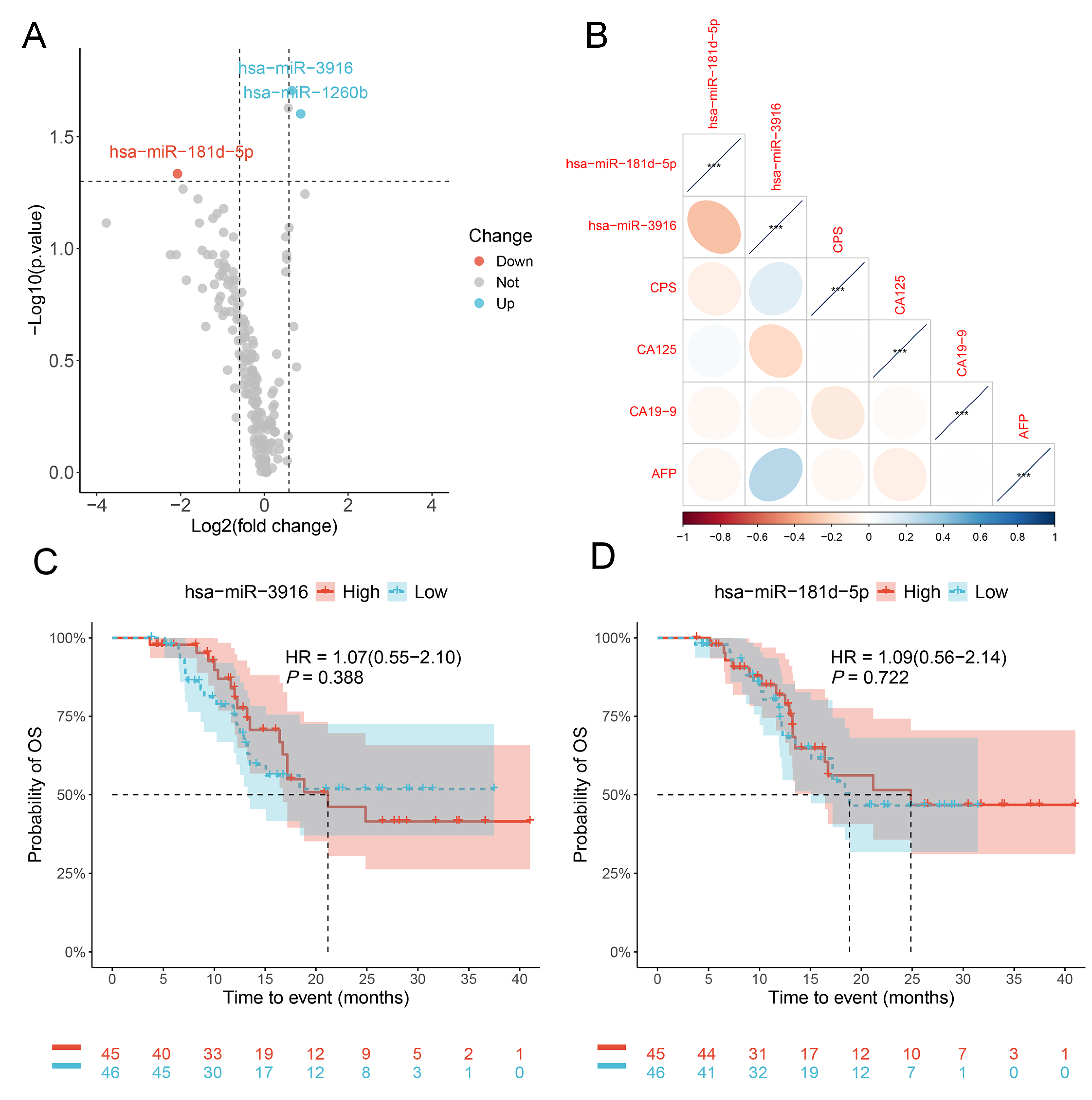


**Supplementary Fig. 2: Clinical outcome prediction by small RNAs in baseline plasma samples of advanced gastric cancer patients.** (**A**) Volcano plot illustrating the baseline plasma sRNAs differentially expressed between responders and non-responders in the discovery cohort. (**B**) Spearman rank correlations between all two sRNAs, PD-L1 CPS, and serological tumor biomarkers (CA125, CA19-9, AFP) in the discovery cohort. An elliptical shape in the grid indicates a significant correlation, with correlation p-values corrected for multiple testing using the false discovery rate (FDR) approach (two-sided rank correlation t-test; FDR ≤ 0.05 cutoff for inclusion in the figure). The size and color intensity of the elliptical shapes reflect the magnitude of the correlation, as indicated in the color legend. Blue elliptical shapes represent positive correlations, while red elliptical shapes signify negative correlations. For each small RNA (sRNA) analyzed, patients with advanced gastric cancer (aGC) were categorized into 'high (H)' or 'low (L)' groups based on the expression levels of the sRNA. The Kaplan-Meier (KM) curve analyses compared overall survival (OS) for (**C**) hsa-miR-3916, and (**D**) hsa-miR-181d-5p. P-values were calculated using the log-rank test. Horizontal and vertical dashed lines indicate median survival time; shaded ribbons represent confidence intervals.


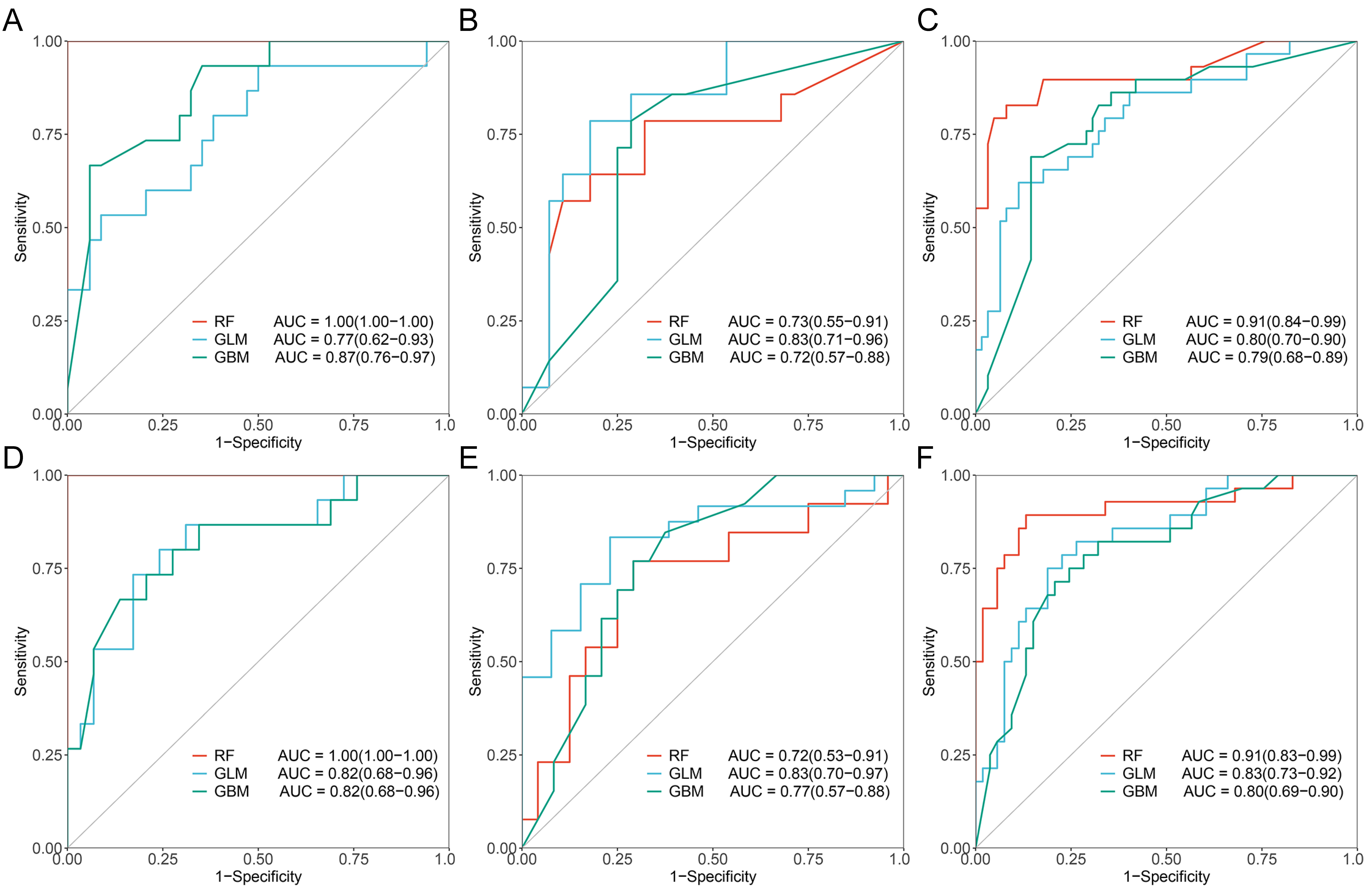


**Supplementary Fig. 3: Classification of responders and non-responders using RF, GLM and GBM models based on plasma small RNA (sRNA) features in advanced gastric cancer patients.** Performance of Random Forest (RF), Generalized Linear Model (GLM), and Stochastic Gradient Boosting (GBM) model using plasma sRNA features to predict responders to combination immunotherapy in the discovery cohort (**A**), the validation cohort (**B**), and the combined dataset (**C**). Performance of RF, GLM, and GBM models using plasma sRNA features combined with PD-L1 CPS positivity to predict responders to combination immunotherapy in the discovery cohort (**D**), the validation cohort (**E**), and the combined dataset (**F**).

**Supplementary Table 1. Tumor response assessed by Response Evaluation Criteria in Solid Tumors (version 1.1).**

|  | Variables | **Number of patients who responded/Total number of patients** | **ORR (95%CI)** | ***p* value** |
| --- | --- | --- | --- | --- |
| **Sex** | | | | |
|  | Male | 44/75 | 58.7 (46.7-69.9) | 0.721 |
|  | Female | 8/16 | 50.0 (24.7-75.3) |  |
| **Histological grade** | | | | |
|  | Poorly differentiated | 20/39 | 51.3 (34.8-67.6) | 0.695 |
|  | Moderately to poorly differentiated | 15/24 | 62.5 (40.6-81.2) |  |
|  | Moderately differentiated | 12/22 | 54.5 (32.2-75.6) |  |
|  | Well to moderately differentiated | 1/1 | 100.0 (2.5-100.0) |  |
|  | Well differentiated | 0/1 | 0.0 (0.0-97.5) |  |
| **Lauren Classification** | | | | |
|  | Diffuse | 12/21 | 57.1 (34.0-78.2) | 0.507 |
|  | Intestinal | 19/38 | 50.0 (33.4-66.6) |  |
|  | Mixed | 15/23 | 65.2 (42.7-83.6) |  |
| **Primary tumor site** | | | | |
|  | Antrum | 18/28 | 64.3 (44.1-81.4) | 0.571 |
|  | Body | 16/32 | 50.0 (31.9-68.1) |  |
|  | Cardia | 17/28 | 60.7 (40.6-78.5) |  |
|  | Fundus | 1/3 | 33.3 (0.8-90.6) |  |
| **ECOG performance status^†^** | | | | |
|  | 0 | 14/23 | 60.9 (38.5-80.3) | 0.862 |
|  | 1 | 38/68 | 55.9 (43.3-67.9) |  |
| **Metastasis site** | | | | |
|  | Liver and lymph node | 13/21 | 61.9 (38.4-81.9) | 0.012 |
|  | Liver only | 15/26 | 57.7 (36.9-76.6) |  |
|  | Lymph node only | 23/34 | 67.6 (49.5-82.6) |  |
|  | Other | 1/10 | 10.0 (0.3-44.5) |  |
| **Number of target lesions** | | | | |
|  | 1 | 25/41 | 61.0 (44.5-75.8) | 0.648 |
|  | 2 or more | 27/50 | 54.0 (39.3-68.2) |  |
| **Lines of therapy** | | | | |
|  | 1L | 49/82 | 59.8 (48.3-70.4) | 0.165 |
|  | 2L | 3/9 | 33.3 (7.5-70.1) |  |
| **PD-L1 status^*^** | | | | |
|  | CPS ＜ 1 | 16/37 | 43.2 (27.1-60.5) | 0.107 |
|  | CPS ≥ 1 | 28/44 | 63.6 (47.8-77.6) |  |
| **MSI status^††^** | | | | |
|  | MSI-H | 2/3 | 66.7 (9.4-99.2) | 0.766 |
|  | MSI-L | 0/1 | 0.0 (0.0-97.5) |  |
|  | MSS | 44/78 | 56.4 (44.7-67.6) |  |
| **Immunotherapy treatment** | | | | |
|  | Combination chemotherapy | 20/44 | 45.5 (30.4-61.2) | 0.049 |
|  | Combination chemotherapy and VEGFR-2 inhibitors | 32/47 | 68.1 (52.9-80.9) |  |
| **Overall** | | | | |
|  |  | 52/91 | 57.1 (46.3-67.5) |  |

^†^ECOG performance status, Eastern Cooperative Oncology Group performance status;

^*^CPS, combined positive score;

^††^MSS, microsatellite stable; MSI, microsatellite instability.

**Supplementary Table 2. Characteristics of combination immunotherapy responses in terms of MSI and PD-L1 IHC.**

| **Patient  ID** | **ECOG performance status** | **Lines of therapy** | **PD-L1  CPS** | **MSI  status^*^** | **Pathology^†^** | **BOR^††^** | **Treatment  response** | **Cohort** |
| --- | --- | --- | --- | --- | --- | --- | --- | --- |
| EP01 | 0 | 1L | N/A | MSS | P/D adneo | PR | Responder | Discovery |
| EP02 | 1 | 1L | 0 | MSS | M-W/D adneo | PR | Responder | Discovery |
| EP03 | 0 | 1L | 0 | MSS | N/A adneo | PR | Responder | Discovery |
| EP04 | 1 | 1L | 0 | MSS | N/A adneo | PR | Responder | Discovery |
| EP05 | 1 | 1L | 2 | MSS | N/A adneo | PR | Responder | Discovery |
| EP06 | 1 | 1L | 10 | MSS | P/D adneo | PR | Responder | Discovery |
| EP07 | 1 | 1L | 0 | MSS | M/D adneo | SD | Non-responder | Discovery |
| EP08 | 0 | 2L | 0 | MSS | P/D adneo | PR | Responder | Discovery |
| EP09 | 1 | 1L | N/A | MSS | P-M/D adneo | PR | Responder | Discovery |
| EP10 | 1 | 2L | 5 | MSS | P-M/D adneo | PR | Responder | Discovery |
| EP11 | 1 | 2L | 5 | MSI-H | M/D adneo | SD | Responder | Discovery |
| EP12 | 1 | 2L | 0 | MSS | M/D adneo | SD | Non-responder | Discovery |
| EP13 | 1 | 2L | 0 | MSS | P/D adneo | SD | Non-responder | Discovery |
| EP14 | 1 | 2L | 0 | MSS | P/D adneo | SD | Responder | Discovery |
| EP15 | 1 | 1L | 1 | MSS | P/D adneo | SD | Non-responder | Discovery |
| EP16 | 1 | 1L | 1 | MSS | P/D adneo | PR | Responder | Discovery |
| EP17 | 1 | 1L | 5 | MSS | P-M/D adneo | PR | Responder | Discovery |
| EP18 | 0 | 1L | 20 | MSS | P/D adneo | PR | Responder | Discovery |
| EP19 | 1 | 1L | N/A | N/A | P-M/D adneo | PR | Responder | Discovery |
| EP20 | 1 | 1L | 2 | MSS | P/D adneo | PD | Non-responder | Discovery |
| EP21 | 1 | 1L | 10 | MSI-H | P-M/D adneo | PR | Responder | Discovery |
| EP22 | 1 | 1L | 5 | MSS | P/D adneo | PR | Responder | Discovery |
| EP23 | 1 | 1L | 2 | MSS | P-M/D adneo | PR | Responder | Discovery |
| EP24 | 0 | 1L | 10 | MSS | P/D adneo | SD | Non-responder | Discovery |
| EP25 | 0 | 1L | N/A | N/A | N/A adneo | PR | Responder | Discovery |
| EP26 | 1 | 1L | 1 | MSS | P-M/D adneo | PR | Responder | Discovery |
| EP27 | 1 | 1L | 2 | N/A | M/D adneo | PR | Responder | Discovery |
| EP28 | 0 | 1L | 0 | MSS | P-M/D adneo | SD | Responder | Discovery |
| EP29 | 1 | 1L | 2 | MSS | P-M/D adneo | SD | Non-responder | Discovery |
| EP30 | 1 | 1L | 0 | MSS | P/D adneo | PR | Responder | Discovery |
| EP31 | 1 | 1L | 3 | MSS | P-M/D adneo | SD | Non-responder | Discovery |
| EP32 | 0 | 1L | 0 | MSS | P/D adneo | PD | Non-responder | Discovery |
| EP33 | 0 | 1L | 1 | MSS | M/D adneo | PR | Responder | Discovery |
| EP34 | 0 | 1L | 8 | MSS | P-M/D adneo | PR | Responder | Discovery |
| EP35 | 1 | 1L | 0 | MSS | P/D adneo | SD | Non-responder | Discovery |
| EP36 | 0 | 1L | 0 | MSS | M/D adneo | PR | Responder | Discovery |
| EP37 | 1 | 1L | 3 | MSS | P/D adneo | SD | Non-responder | Discovery |
| EP38 | 1 | 1L | 0 | MSS | P/D adneo | SD | Non-responder | Discovery |
| EP39 | 1 | 1L | N/A | MSS | P/D adneo | PR | Responder | Discovery |
| EP40 | 1 | 1L | 1 | MSS | P/D adneo | SD | Responder | Discovery |
| EP41 | 1 | 1L | 0 | MSS | P-M/D adneo | PR | Responder | Discovery |
| EP42 | 1 | 1L | 2 | MSS | P-M/D adneo | PR | Responder | Discovery |
| EP43 | 1 | 2L | 0 | MSS | M/D adneo | SD | Non-responder | Discovery |
| EP44 | 1 | 2L | 0 | MSS | P-M/D adneo | PD | Non-responder | Discovery |
| EP45 | 1 | 1L | 0 | MSI-L | M/D adneo | PD | Non-responder | Discovery |
| EP46 | 1 | 1L | 1 | MSS | P/D adneo | PR | Responder | Discovery |
| EP47 | 0 | 2L | 3 | MSS | P-M/D adneo | PR | Responder | Discovery |
| EP48 | 1 | 1L | 2 | MSS | M/D adneo | PR | Responder | Discovery |
| EP49 | 1 | 1L | 5 | MSS | P/D adneo | SD | Responder | Discovery |
| EP50 | 1 | 1L | 4 | MSI-H | P-M/D adneo | PR | Responder | Validation |
| EP51 | 0 | 1L | 1 | MSS | M/D adneo | PR | Responder | Validation |
| EP52 | 0 | 1L | N/A | MSS | P/D adneo | PR | Responder | Validation |
| EP54 | 1 | 1L | 2 | MSS | M/D adneo | PR | Responder | Validation |
| EP55 | 0 | 1L | N/A | MSS | P-M/D adneo | PR | Responder | Validation |
| EP59 | 1 | 1L | N/A | MSS | P/D adneo | SD | Responder | Validation |
| EP60 | 1 | 1L | 0 | MSS | P/D adneo | PR | Responder | Validation |
| EP61 | 1 | 1L | 0 | MSS | P/D adneo | SD | Responder | Validation |
| EP62 | 1 | 1L | 0 | MSS | M/D adneo | PR | Responder | Validation |
| EP63 | 1 | 1L | 0 | MSS | M/D adneo | PR | Responder | Validation |
| EP64 | 1 | 1L | 0 | MSS | M/D adneo | PR | Responder | Validation |
| EP66 | 1 | 1L | 0 | MSS | P/D adneo | SD | Non-responder | Validation |
| EP68 | 1 | 1L | 0 | MSS | P/D adneo | PR | Responder | Validation |
| EP69 | 0 | 1L | 1 | MSS | P-M/D adneo | SD | Responder | Validation |
| EP70 | 1 | 1L | 0 | MSS | P-M/D adneo | PD | Non-responder | Validation |
| EP71 | 1 | 1L | 0 | MSS | P/D adneo | PR | Responder | Validation |
| EP72 | 1 | 1L | 0 | MSS | P/D adneo | SD | Non-responder | Validation |
| EP73 | 1 | 1L | 0 | MSS | P-M/D adneo | SD | Non-responder | Validation |
| EP74 | 1 | 1L | 1 | MSS | P-M/D adneo | PR | Responder | Validation |
| EP75 | 0 | 1L | 1 | MSS | M/D adneo | PD | Non-responder | Validation |
| EP76 | 1 | 1L | 0 | MSS | M/D adneo | PR | Responder | Validation |
| EP77 | 1 | 1L | N/A | N/A | P/D adneo | SD | Non-responder | Validation |
| EP78 | 1 | 1L | 15 | MSS | P/D adneo | PR | Responder | Validation |
| EP79 | 0 | 1L | 3 | MSS | P/D adneo | PR | Responder | Validation |
| EP80 | 0 | 1L | 1 | MSS | P/D adneo | SD | Responder | Validation |
| EP81 | 1 | 1L | 1 | MSS | M/D adneo | PD | Non-responder | Validation |
| EP82 | 0 | 1L | 10 | MSS | P-M/D adneo | SD | Responder | Validation |
| EP83 | 1 | 1L | 1 | MSS | M/D adneo | SD | Non-responder | Validation |
| EP84 | 1 | 1L | 0 | MSS | M/D adneo | SD | Non-responder | Validation |
| EP85 | 1 | 1L | 0 | MSS | P/D adneo | SD | Non-responder | Validation |
| EP86 | 1 | 1L | 0 | MSS | P-M/D adneo | SD | Non-responder | Validation |
| EP87 | 1 | 1L | 2 | MSS | P-M/D adneo | PR | Responder | Validation |
| EP89 | 0 | 1L | 3 | MSS | P/D adneo | PR | Responder | Validation |
| EP90 | 1 | 1L | 2 | MSS | P/D adneo | PR | Responder | Validation |
| EP91 | 1 | 1L | 0 | MSS | M/D adneo | PR | Responder | Validation |
| EP92 | 0 | 1L | 0 | N/A | M/D adneo | PD | Non-responder | Validation |
| EP93 | 1 | 1L | N/A | N/A | P/D adneo | PR | Responder | Validation |
| EP94 | 1 | 1L | 0 | N/A | P/D adneo | PR | Responder | Validation |
| EP95 | 1 | 1L | 2 | N/A | M/D adneo | PR | Responder | Validation |
| EP96 | 1 | 1L | 0 | N/A | W/D adneo | PD | Non-responder | Validation |
| EP97 | 0 | 1L | 1 | MSS | P/D adneo | SD | Non-responder | Validation |
| EP99 | 1 | 1L | 5 | MSS | P/D adneo | PR | Responder | Validation |

*MSS, microsatellite stable; MSI, microsatellite instability;

†P/D, poorly differentiated; M/D, moderately differentiated; P-M/D, poorly-moderately differentiated adenocarcinoma;

††BOR, best of response; CR, complete response; PR, partial response; SD, stable disease; PD, progressive disease;

ID, identity;

N/A, not available.

**Supplementary Table 3. Summary of patients’ clinical information in discovery and validation cohorts.**

| **Patient  ID** | **Treatment  response** | **Cohort** | **Treatment** | **PLR^*^** | **NLR^†^** | **SII^††^** | **CEA (ng/ml)** | **CA72-4 (U/ml)** | **CA125 (U/ml)** | **CA19-9 (U/ml)** | **AFP (ng/ml)** | **Tumor size (mm)** |
| --- | --- | --- | --- | --- | --- | --- | --- | --- | --- | --- | --- | --- |
| EP01 | Responder | Discovery | Pre-treatment | 555.29 | 4.56 | 2154.54 | 15.50 | 1.50 | 24.00 | 128.00 | 121000.00 | 125.40 |
| EP02 | Responder | Discovery | Pre-treatment | 141.67 | 3.07 | 365.50 | 2.02 | 6.58 | 15.10 | 55.35 | 2.50 | 65.00 |
| EP03 | Responder | Discovery | Pre-treatment | 76.23 | 2.39 | 221.83 | 4.27 | 3.50 | 8.70 | 0.60 | 545.80 | 48.68 |
| EP04 | Responder | Discovery | Pre-treatment | 116.58 | 1.96 | 455.84 | 69.55 | 2.52 | 12.60 | 1000.00 | 3.08 | 82.31 |
| EP05 | Responder | Discovery | Pre-treatment | 188.00 | 5.81 | 819.68 | 48.52 | 69.24 | 25.60 | 8.08 | 1.25 | 33.42 |
| EP06 | Responder | Discovery | Pre-treatment | 77.19 | 2.90 | 255.51 | 711.00 | 1.63 | 56.80 | 11.10 | 2.46 | 77.10 |
| EP07 | Non-responder | Discovery | Pre-treatment | 198.44 | 1.47 | 373.06 | 89.40 | 33.20 | 19.20 | 30417.00 | 2653.00 | 27.80 |
| EP08 | Responder | Discovery | Pre-treatment | 266.96 | 2.99 | 894.33 | 1.79 | 96.80 | 19.60 | 15.30 | 3.13 | 15.20 |
| EP09 | Responder | Discovery | Pre-treatment | 204.37 | 3.16 | 1183.31 | 39.40 | 9.72 | 71.80 | 11.01 | 7.78 | 52.27 |
| EP10 | Responder | Discovery | Pre-treatment | 91.86 | 0.84 | 132.28 | 3.93 | 2.34 | 12.10 | 90.20 | 2.91 | 15.10 |
| EP11 | Responder | Discovery | Pre-treatment | 123.58 | 3.70 | 484.45 | 3.43 | 1.95 | 11.50 | 7.01 | 2.43 | 16.20 |
| EP12 | Non-responder | Discovery | Pre-treatment | 77.61 | 2.27 | 354.69 | 276.00 | 8.85 | 10.50 | 22.10 | 2.16 | 47.40 |
| EP13 | Non-responder | Discovery | Pre-treatment | 136.30 | 2.65 | 487.94 | 2.40 | 2.55 | 4.28 | 11.40 | 2.79 | 15.90 |
| EP14 | Responder | Discovery | Pre-treatment | 128.83 | 1.78 | 373.62 | 2.62 | 9.84 | 9.22 | 173.00 | 2.43 | 50.60 |
| EP15 | Non-responder | Discovery | Pre-treatment | 333.33 | 5.78 | 1040.00 | 10.07 | 1.81 | 83.60 | 3.69 | 1210.00 | 126.93 |
| EP16 | Responder | Discovery | Pre-treatment | 57.05 | 3.51 | 312.64 | 0.97 | 2.30 | 15.80 | 8.18 | 3.98 | 11.35 |
| EP17 | Responder | Discovery | Pre-treatment | 186.27 | 1.61 | 460.10 | 3.27 | 1.50 | 30.10 | 32.50 | 2.52 | 15.00 |
| EP18 | Responder | Discovery | Pre-treatment | 233.33 | 5.97 | 1087.33 | 6.18 | 0.89 | 13.50 | 271.40 | 3.81 | 121.30 |
| EP19 | Responder | Discovery | Pre-treatment | 76.67 | 1.54 | 319.70 | 1.64 | 1.50 | 8.16 | 8.29 | 2.82 | 15.20 |
| EP20 | Non-responder | Discovery | Pre-treatment | 206.19 | 3.24 | 1294.85 | 3.73 | 17.80 | 48.50 | 494.00 | 3.85 | 11.30 |
| EP21 | Responder | Discovery | Pre-treatment | 268.61 | 1.98 | 727.94 | 2.16 | 24.50 | 9.71 | 5.08 | 3.45 | 54.40 |
| EP22 | Responder | Discovery | Pre-treatment | 178.77 | 1.38 | 441.56 | 0.75 | 173.00 | 156.00 | 244.00 | 5.18 | 15.10 |
| EP23 | Responder | Discovery | Pre-treatment | 135.36 | 3.69 | 902.85 | 179.00 | 53.10 | 93.20 | 3313.00 | 191.00 | 65.00 |
| EP24 | Non-responder | Discovery | Pre-treatment | 153.01 | 3.34 | 847.69 | 0.57 | 84.60 | 17.60 | 5.44 | 1.36 | 19.00 |
| EP25 | Responder | Discovery | Pre-treatment | 120.51 | 3.52 | 661.62 | 2.03 | 2.48 | 21.10 | 1.24 | 183.40 | 98.10 |
| EP26 | Responder | Discovery | Pre-treatment | 414.17 | 4.65 | 2447.76 | 3.00 | 4.97 | 39.40 | 169.90 | 2.12 | 51.40 |
| EP27 | Responder | Discovery | Pre-treatment | 152.38 | 1.85 | 591.24 | 11.30 | 3.65 | 15.10 | 2.00 | 4911.00 | 64.10 |
| EP28 | Responder | Discovery | Pre-treatment | 204.29 | 4.07 | 1164.43 | 1.57 | 250.00 | 15.10 | 176.76 | 3.80 | 61.40 |
| EP29 | Non-responder | Discovery | Pre-treatment | 205.77 | 5.56 | 1189.35 | 16.90 | 3.92 | 7.60 | 15.40 | 66444.00 | 72.10 |
| EP30 | Responder | Discovery | Pre-treatment | 343.75 | 10.00 | 1100.00 | 1.27 | 1.01 | 17.00 | 9.50 | 2.40 | 12.20 |
| EP31 | Non-responder | Discovery | Pre-treatment | 358.14 | 6.77 | 2084.37 | 10.50 | 12.20 | 351.00 | 19.30 | 2.95 | 13.60 |
| EP32 | Non-responder | Discovery | Pre-treatment | 300.00 | 3.45 | 870.00 | 1.42 | 6.81 | 51.90 | 5.62 | 1210.00 | 142.30 |
| EP33 | Responder | Discovery | Pre-treatment | 144.23 | 4.76 | 1070.19 | 5.73 | 7.59 | 9.00 | 17.08 | 5.69 | 25.70 |
| EP34 | Responder | Discovery | Pre-treatment | 103.79 | 3.34 | 457.70 | 2.89 | 1.36 | 7.60 | 8.40 | 3.51 | 16.10 |
| EP35 | Non-responder | Discovery | Pre-treatment | 136.30 | 2.65 | 487.94 | 168.00 | 1.50 | 16.00 | 130.00 | 1.53 | 105.00 |
| EP36 | Responder | Discovery | Pre-treatment | 117.19 | 3.72 | 837.89 | 175.00 | 8.06 | 9.50 | 983.00 | 324.40 | 39.60 |
| EP37 | Non-responder | Discovery | Pre-treatment | 137.32 | 1.97 | 384.51 | 2.20 | 5.29 | 16.40 | 6.31 | 2.84 | 27.00 |
| EP38 | Non-responder | Discovery | Pre-treatment | 314.49 | 3.17 | 1374.33 | 28.10 | 60.80 | 153.00 | 804.00 | 2.31 | 16.00 |
| EP39 | Responder | Discovery | Pre-treatment | 156.25 | 4.89 | 610.94 | 2.03 | 6.58 | 12.30 | 2.72 | 2.92 | 29.83 |
| EP40 | Responder | Discovery | Pre-treatment | 335.59 | 1.33 | 526.88 | 1.38 | 8.21 | 48.60 | 2.00 | 1.45 | 48.60 |
| EP41 | Responder | Discovery | Pre-treatment | 205.00 | 1.66 | 475.60 | 2.04 | 30.50 | 35.10 | 2.00 | 1.47 | 15.50 |
| EP42 | Responder | Discovery | Pre-treatment | 150.88 | 1.54 | 264.04 | 13.10 | 1.50 | 55.10 | 9.99 | 37.20 | 25.00 |
| EP43 | Non-responder | Discovery | Pre-treatment | 143.93 | 3.07 | 472.07 | 56.20 | 1.65 | 13.50 | 56.20 | 4384.00 | 46.60 |
| EP44 | Non-responder | Discovery | Pre-treatment | 101.27 | 3.77 | 602.53 | 272.00 | 3.86 | 29.00 | 28.40 | 51.20 | 113.40 |
| EP45 | Non-responder | Discovery | Pre-treatment | 126.44 | 7.06 | 776.32 | 60.92 | 124.90 | 19.20 | 1000.00 | 15.93 | 99.60 |
| EP46 | Responder | Discovery | Pre-treatment | 345.28 | 5.96 | 1091.09 | 11.85 | 2.30 | 18.80 | 6.77 | 3.86 | 15.02 |
| EP47 | Responder | Discovery | Pre-treatment | 106.05 | 1.79 | 408.28 | 1.47 | 2.08 | 14.10 | 7.57 | 3.24 | 27.90 |
| EP48 | Responder | Discovery | Pre-treatment | 128.49 | 2.54 | 584.64 | 9.84 | 34.00 | 11.20 | 112.00 | 2.97 | 54.00 |
| EP49 | Responder | Discovery | Pre-treatment | 122.09 | 1.93 | 405.35 | 1.76 | 9.36 | 6.20 | 22.47 | 4.39 | 41.99 |
| EP50 | Responder | Validation | Pre-treatment | 152.86 | 2.34 | 501.37 | 0.20 | 91.67 | 115.40 | 13.71 | 2.07 | 39.10 |
| EP51 | Responder | Validation | Pre-treatment | 134.57 | 6.52 | 1421.04 | 15.73 | 797.40 | 13.00 | 1392.00 | 0.90 | 158.51 |
| EP52 | Responder | Validation | Pre-treatment | 329.17 | 6.79 | 536.54 | 104.60 | 10.95 | 15.00 | 36.49 | 1.69 | 19.56 |
| EP54 | Responder | Validation | Pre-treatment | 215.60 | 9.34 | 2194.77 | 243.50 | 9.72 | 15.10 | 31.73 | 23.58 | 87.24 |
| EP55 | Responder | Validation | Pre-treatment | 222.67 | 5.68 | 948.56 | 5.70 | 4.73 | 15.10 | 55.12 | 1.43 | 51.96 |
| EP59 | Responder | Validation | Pre-treatment | 222.67 | 3.13 | 1046.53 | 1021.00 | 8.82 | 277.00 | 1000.00 | 2.82 | 23.00 |
| EP60 | Responder | Validation | Pre-treatment | 91.84 | 3.09 | 278.27 | 2.93 | 1.43 | 16.30 | 25.73 | 42.35 | 45.67 |
| EP61 | Responder | Validation | Pre-treatment | 197.60 | 2.07 | 683.71 | 13.82 | 4.65 | 73.78 | 7.15 | 22.74 | 38.00 |
| EP62 | Responder | Validation | Pre-treatment | 175.94 | 1.74 | 408.18 | 123.00 | 7.90 | 0.00 | 15.46 | 6.70 | 34.00 |
| EP63 | Responder | Validation | Pre-treatment | 535.59 | 22.93 | 7246.58 | 35.18 | 5.63 | 22.70 | 63.32 | 2.75 | 189.71 |
| EP64 | Responder | Validation | Pre-treatment | 58.71 | 0.83 | 151.47 | 13.72 | 4.44 | 10.80 | 306.70 | 1.14 | 26.48 |
| EP66 | Non-responder | Validation | Pre-treatment | 152.73 | 5.08 | 853.75 | 30.68 | 4.30 | 16.10 | 1.13 | 3.01 | 93.68 |
| EP68 | Responder | Validation | Pre-treatment | 149.04 | 1.01 | 391.98 | 1.32 | 6.58 | 1.00 | 5.44 | 1.43 | 18.00 |
| EP69 | Responder | Validation | Pre-treatment | 51.34 | 0.61 | 106.26 | 3.57 | 1.60 | 27.70 | 31.31 | 3.50 | 35.81 |
| EP70 | Non-responder | Validation | Pre-treatment | 170.83 | 13.08 | 1072.83 | 73.80 | 120.00 | 14.80 | 1380.00 | 3.42 | 85.00 |
| EP71 | Responder | Validation | Pre-treatment | 249.09 | 2.35 | 963.98 | 1.71 | 0.81 | 137.40 | 10.41 | 3.18 | 82.69 |
| EP72 | Non-responder | Validation | Pre-treatment | 159.62 | 3.04 | 504.38 | 2.38 | 1.39 | 17.00 | 83.38 | 1.21 | 15.76 |
| EP73 | Non-responder | Validation | Pre-treatment | 93.41 | 1.31 | 203.64 | 231.00 | 2.93 | 25.10 | 14.40 | 2.60 | 65.80 |
| EP74 | Responder | Validation | Pre-treatment | 117.05 | 2.49 | 375.74 | 1.28 | 1.89 | 20.90 | 75.30 | 3.86 | 18.70 |
| EP75 | Non-responder | Validation | Pre-treatment | 183.52 | 3.16 | 1057.05 | 336.00 | 537.00 | 51.40 | 100000.00 | 4.17 | 90.50 |
| EP76 | Responder | Validation | Pre-treatment | 307.89 | 4.56 | 1601.05 | 15.00 | 50.60 | 13.50 | 380.00 | 127.00 | 44.30 |
| EP77 | Non-responder | Validation | Pre-treatment | 203.31 | 3.02 | 744.10 | 378.00 | 240.00 | 66.50 | 18.30 | 3.79 | 15.80 |
| EP78 | Responder | Validation | Pre-treatment | 188.52 | 2.90 | 667.38 | 5.56 | 42.90 | 6.45 | 5.50 | 2.38 | 18.00 |
| EP79 | Responder | Validation | Pre-treatment | 246.56 | 2.65 | 855.58 | 2.21 | 21.53 | 37.30 | 8.53 | 4.04 | 10.08 |
| EP80 | Responder | Validation | Pre-treatment | 204.26 | 2.20 | 422.81 | 25.60 | 300.00 | 10.90 | 15.78 | 1.91 | 27.42 |
| EP81 | Non-responder | Validation | Pre-treatment | 263.46 | 4.63 | 1269.88 | 5.26 | 1.52 | 27.70 | 5.45 | 1.49 | 152.81 |
| EP82 | Responder | Validation | Pre-treatment | 217.16 | 3.66 | 1066.28 | 264.00 | 141.00 | 49.80 | 374.00 | 2.51 | 43.00 |
| EP83 | Non-responder | Validation | Pre-treatment | 58.59 | 9.54 | 1106.10 | 16.58 | 2.00 | 19.20 | 22.97 | 11.05 | 103.92 |
| EP84 | Non-responder | Validation | Pre-treatment | 188.24 | 2.10 | 269.18 | 7.32 | 2.75 | 4.84 | 9.77 | 154.00 | 15.00 |
| EP85 | Non-responder | Validation | Pre-treatment | 168.97 | 1.44 | 211.21 | 3.21 | 1.69 | 16.30 | 6.85 | 2.93 | 57.00 |
| EP86 | Non-responder | Validation | Pre-treatment | 224.32 | 3.85 | 1278.65 | 16471.00 | 11.80 | 106.00 | 2.00 | 294.00 | 91.20 |
| EP87 | Responder | Validation | Pre-treatment | 173.39 | 1.31 | 247.95 | 2.05 | 10.50 | 11.50 | 24.90 | 4.80 | 37.00 |
| EP89 | Responder | Validation | Pre-treatment | 201.53 | 2.86 | 755.73 | 10.14 | 36.14 | 23.30 | 4.40 | 152.30 | 71.98 |
| EP90 | Responder | Validation | Pre-treatment | 198.45 | 3.05 | 781.89 | 1543.00 | 542.00 | 4.15 | 515.00 | 3.98 | 70.30 |
| EP91 | Responder | Validation | Pre-treatment | 134.02 | 2.81 | 365.88 | 2.28 | 1.60 | 6.40 | 11.94 | 1.14 | 13.40 |
| EP92 | Non-responder | Validation | Pre-treatment | 236.36 | 4.34 | 1581.27 | 2.71 | 22.47 | 9.30 | 2.60 | 1.13 | 30.53 |
| EP93 | Responder | Validation | Pre-treatment | 141.14 | 2.46 | 549.03 | 26.62 | 9.42 | 20.50 | 4.72 | 2.44 | 15.34 |
| EP94 | Responder | Validation | Pre-treatment | 130.83 | 2.44 | 425.19 | 17.28 | 1.29 | 6.40 | 3.58 | 192.40 | 41.56 |
| EP95 | Responder | Validation | Pre-treatment | 130.77 | 2.24 | 419.77 | 2.88 | 2.70 | 10.90 | 608.90 | 2.03 | 112.85 |
| EP96 | Non-responder | Validation | Pre-treatment | 166.41 | 1.95 | 416.02 | 32.25 | 102.00 | 87.41 | 1000.00 | 3.13 | 38.88 |
| EP97 | Non-responder | Validation | Pre-treatment | 148.05 | 3.49 | 661.77 | 1.61 | 8.32 | 21.00 | 272.00 | 3.75 | 33.30 |
| EP99 | Responder | Validation | Pre-treatment | 167.67 | 2.66 | 593.55 | 10.90 | 6.58 | 18.90 | 139.00 | 2.59 | 48.10 |
| EP01 | Responder | Discovery | Post-treatment | 114.86 | 1.11 | 283.72 | 14.20 | 4.22 | 7.21 | 58.80 | 4359.00 | 89.60 |
| EP02 | Responder | Discovery | Post-treatment | 137.70 | 11.38 | 955.67 | 2.27 | 5.24 | 9.90 | 43.36 | 1.91 | 25.50 |
| EP03 | Responder | Discovery | Post-treatment | 91.89 | 4.39 | 298.65 | 3.39 | 12.20 | 9.28 | 0.93 | 6.38 | 21.66 |
| EP04 | Responder | Discovery | Post-treatment | 120.61 | 2.30 | 457.10 | 13.00 | 1.06 | 7.70 | 150.10 | 2.91 | 52.51 |
| EP05 | Responder | Discovery | Post-treatment | 366.67 | 26.74 | 2647.33 | 3.09 | 11.02 | 11.20 | 6.79 | 1.68 | 11.41 |
| EP06 | Responder | Discovery | Post-treatment | 168.00 | 3.68 | 463.68 | 34.90 | 2.00 | 41.90 | 12.60 | 1.07 | 33.00 |
| EP07 | Non-responder | Discovery | Post-treatment | 104.14 | 2.22 | 391.57 | 70.10 | 5.39 | 9.93 | 34212.00 | 688.00 | 14.20 |
| EP08 | Responder | Discovery | Post-treatment | 268.03 | 2.20 | 718.33 | 1.05 | 24.10 | 16.30 | 14.30 | 2.74 | 5.00 |
| EP09 | Responder | Discovery | Post-treatment | 114.38 | 1.39 | 255.06 | 5.08 | 1.08 | 3.60 | 13.00 | 3.24 | 20.66 |
| EP10 | Responder | Discovery | Post-treatment | 168.29 | 4.73 | 979.46 | 3.29 | 1.99 | 8.56 | 15.60 | 1.10 | 7.90 |
| EP11 | Responder | Discovery | Post-treatment | 94.12 | 3.26 | 416.94 | 2.78 | 4.40 | 7.67 | 4.01 | 2.73 | 13.00 |
| EP12 | Non-responder | Discovery | Post-treatment | 93.06 | 2.38 | 319.18 | 309.00 | 8.40 | 9.86 | 19.80 | 2.35 | 48.00 |
| EP13 | Non-responder | Discovery | Post-treatment | 169.77 | 2.58 | 565.33 | 3.37 | 1.50 | 6.00 | 17.50 | 4.38 | 15.00 |
| EP14 | Responder | Discovery | Post-treatment | 143.08 | 3.05 | 568.02 | 2.58 | 11.40 | 9.26 | 164.00 | 2.73 | 54.30 |
| EP15 | Non-responder | Discovery | Post-treatment | 63.52 | 1.30 | 130.86 | 16.44 | 6.99 | 28.20 | 25.50 | 100672.00 | 92.62 |
| EP16 | Responder | Discovery | Post-treatment | 112.37 | 6.42 | 700.07 | 1.45 | 3.68 | 8.00 | 8.40 | 2.27 | 6.89 |
| EP17 | Responder | Discovery | Post-treatment | 86.13 | 1.20 | 179.14 | 3.48 | 1.50 | 15.10 | 37.40 | 2.49 | 8.10 |
| EP18 | Responder | Discovery | Post-treatment | 196.43 | 4.29 | 471.43 | 2.60 | 0.99 | 6.70 | 35.27 | 3.86 | 63.30 |
| EP19 | Responder | Discovery | Post-treatment | 73.19 | 1.85 | 428.87 | 1.30 | 3.62 | 13.20 | 10.30 | 3.33 | 10.00 |
| EP20 | Non-responder | Discovery | Post-treatment | 132.46 | 1.82 | 460.96 | 4.85 | 48.30 | 171.00 | 200.00 | 3.09 | 12.20 |
| EP21 | Responder | Discovery | Post-treatment | 135.66 | 1.82 | 318.80 | 3.03 | 1.79 | 11.20 | 7.17 | 2.54 | 36.30 |
| EP22 | Responder | Discovery | Post-treatment | 95.87 | 2.02 | 234.88 | 1.41 | 4.12 | 9.26 | 17.00 | 4.28 | 5.40 |
| EP23 | Responder | Discovery | Post-treatment | 85.19 | 1.80 | 165.26 | 13.20 | 14.30 | 23.90 | 114.00 | 8.98 | 22.30 |
| EP24 | Non-responder | Discovery | Post-treatment | 62.50 | 1.98 | 168.13 | 0.59 | 25.30 | 22.80 | 11.00 | 3.80 | 18.50 |
| EP25 | Responder | Discovery | Post-treatment | 100.70 | 4.51 | 644.51 | 2.54 | 3.67 | 10.50 | 0.98 | 6.20 | 51.40 |
| EP26 | Responder | Discovery | Post-treatment | 220.00 | 9.36 | 1441.00 | 2.58 | 1.19 | 8.90 | 21.40 | 4.55 | 21.20 |
| EP27 | Responder | Discovery | Post-treatment | 133.53 | 1.26 | 280.42 | 3.08 | 3.23 | 11.70 | 2.00 | 3.55 | 32.90 |
| EP28 | Responder | Discovery | Post-treatment | 78.57 | 1.33 | 220.00 | 2.06 | 213.00 | 9.28 | 246.20 | 4.90 | 46.60 |
| EP29 | Non-responder | Discovery | Post-treatment | 88.71 | 1.90 | 208.47 | 24.20 | 51.70 | 10.20 | 29.00 | 3174.00 | 70.40 |
| EP30 | Responder | Discovery | Post-treatment | 205.26 | 30.68 | 1196.68 | 1.93 | 1.17 | 14.60 | 18.78 | 3.84 | 5.00 |
| EP31 | Non-responder | Discovery | Post-treatment | 198.90 | 4.56 | 825.44 | 6.00 | 14.30 | 92.20 | 8.85 | 2.82 | 10.60 |
| EP32 | Non-responder | Discovery | Post-treatment | 295.24 | 6.07 | 1505.71 | 2.18 | 1.61 | 130.20 | 281.00 | 17696.00 | 250.80 |
| EP33 | Responder | Discovery | Post-treatment | 85.79 | 1.36 | 212.77 | 9.81 | 2.55 | 8.10 | 58.78 | 4.05 | 13.20 |
| EP34 | Responder | Discovery | Post-treatment | 73.23 | 0.68 | 98.86 | 8.58 | 1.06 | 10.40 | 14.93 | 4.28 | 5.00 |
| EP35 | Non-responder | Discovery | Post-treatment | 100.69 | 2.65 | 386.65 | 552.00 | 1.50 | 45.50 | 141.00 | 3.38 | 119.10 |
| EP36 | Responder | Discovery | Post-treatment | 38.58 | 0.87 | 85.27 | 122.60 | 8.27 | 6.90 | 1000.00 | 11.01 | 20.10 |
| EP37 | Non-responder | Discovery | Post-treatment | 140.26 | 1.65 | 356.26 | 3.46 | 3.12 | 13.10 | 5.70 | 2.80 | 26.50 |
| EP38 | Non-responder | Discovery | Post-treatment | 239.57 | 1.53 | 510.28 | 25.00 | 45.90 | 82.20 | 491.00 | 3.50 | 13.10 |
| EP39 | Responder | Discovery | Post-treatment | 108.82 | 2.49 | 368.91 | 2.12 | 1.17 | 8.60 | 2.97 | 2.43 | 11.73 |
| EP40 | Responder | Discovery | Post-treatment | 186.72 | 1.59 | 380.91 | 2.04 | 30.50 | 35.10 | 2.00 | 1.47 | 42.50 |
| EP41 | Responder | Discovery | Post-treatment | 88.10 | 1.52 | 225.52 | 2.64 | 21.30 | 14.50 | 1405.00 | 3.45 | 10.00 |
| EP42 | Responder | Discovery | Post-treatment | 62.35 | 1.01 | 53.62 | 4.37 | 1.50 | 26.50 | 8.47 | 5.07 | 8.60 |
| EP43 | Non-responder | Discovery | Post-treatment | 118.26 | 2.46 | 334.68 | 76.30 | 1.50 | 15.30 | 41.20 | 5355.00 | 47.50 |
| EP44 | Non-responder | Discovery | Post-treatment | 106.61 | 6.70 | 864.62 | 123.00 | 3.83 | 42.00 | 22.90 | 33.30 | 109.40 |
| EP45 | Non-responder | Discovery | Post-treatment | 62.25 | 0.83 | 78.44 | 27.12 | 11.37 | 58.60 | 1000.00 | 5.61 | 65.20 |
| EP46 | Responder | Discovery | Post-treatment | 148.78 | 2.04 | 248.46 | 5.40 | 1.95 | 10.00 | 9.89 | 3.05 | 10.21 |
| EP47 | Responder | Discovery | Post-treatment | 224.18 | 2.42 | 829.48 | 1.20 | 2.01 | 10.30 | 5.43 | 2.77 | 24.70 |
| EP48 | Responder | Discovery | Post-treatment | 70.43 | 1.55 | 203.54 | 4.66 | 2.56 | 11.00 | 23.50 | 2.86 | 19.20 |
| EP49 | Responder | Discovery | Post-treatment | 116.91 | 1.18 | 188.23 | 1.05 | 10.33 | 3.80 | 13.50 | 3.45 | 39.90 |
| EP50 | Responder | Validation | Post-treatment | 106.67 | 1.38 | 198.40 | 0.74 | 5.33 | 290.80 | 20.34 | 3.32 | 26.30 |
| EP51 | Responder | Validation | Post-treatment | 84.34 | 5.41 | 378.67 | 4.22 | 14.48 | 8.60 | 34.58 | 1.22 | 97.66 |
| EP52 | Responder | Validation | Post-treatment | 81.54 | 6.88 | 364.48 | 4.31 | 0.97 | 4.40 | 6.91 | 0.65 | 11.32 |
| EP54 | Responder | Validation | Post-treatment | 143.84 | 4.55 | 956.51 | 232.20 | 5.29 | 9.28 | 44.26 | 2.78 | 53.64 |
| EP55 | Responder | Validation | Post-treatment | 265.96 | 2.74 | 343.09 | 2.01 | 10.30 | 7.65 | 1.55 | 3.28 | 30.76 |
| EP59 | Responder | Validation | Post-treatment | 364.29 | 4.00 | 1020.00 | 1033.00 | 5.47 | 115.00 | 1000.00 | 2.48 | 23.00 |
| EP60 | Responder | Validation | Post-treatment | 241.94 | 3.84 | 287.90 | 2.45 | 1.32 | 20.90 | 9.13 | 49.92 | 18.48 |
| EP61 | Responder | Validation | Post-treatment | 125.68 | 2.04 | 470.05 | 21.27 | 4.12 | 9.28 | 14.20 | 3.28 | 26.00 |
| EP62 | Responder | Validation | Post-treatment | 1.20 | 0.01 | 2.09 | 40.53 | 7.55 | 9.28 | 8.40 | 2.50 | 19.00 |
| EP63 | Responder | Validation | Post-treatment | 142.20 | 1.09 | 267.33 | 2.43 | 1.75 | 23.70 | 36.44 | 3.56 | 117.59 |
| EP64 | Responder | Validation | Post-treatment | 29.12 | 0.93 | 71.05 | 2.87 | 1.14 | 7.60 | 33.25 | 1.29 | 12.14 |
| EP66 | Non-responder | Validation | Post-treatment | 130.36 | 4.04 | 589.21 | 6.48 | 16.38 | 28.20 | 1.14 | 2.56 | 51.39 |
| EP68 | Responder | Validation | Post-treatment | 51.34 | 0.45 | 77.00 | 3.81 | 1.54 | 0.00 | 10.20 | 1.88 | 6.00 |
| EP69 | Responder | Validation | Post-treatment | 70.91 | 0.91 | 106.36 | 8.46 | 6.92 | 8.30 | 15.30 | 7.49 | 23.12 |
| EP70 | Non-responder | Validation | Post-treatment | 49.06 | 25.04 | 650.98 | 194.00 | 168.00 | 110.00 | 4382.00 | 1.97 | 119.00 |
| EP71 | Responder | Validation | Post-treatment | 171.43 | 1.35 | 372.00 | 3.52 | 0.94 | 11.80 | 13.83 | 3.64 | 16.00 |
| EP72 | Non-responder | Validation | Post-treatment | 104.81 | 1.78 | 193.89 | 7.68 | 1.72 | 65.80 | 58.28 | 0.86 | 14.37 |
| EP73 | Non-responder | Validation | Post-treatment | 84.28 | 0.97 | 129.79 | 144.00 | 15.20 | 21.20 | 9.18 | 2.92 | 62.00 |
| EP74 | Responder | Validation | Post-treatment | 45.24 | 1.59 | 90.48 | 5.33 | 2.32 | 9.42 | 18.40 | 5.75 | 11.00 |
| EP75 | Non-responder | Validation | Post-treatment | 54.40 | 2.38 | 235.53 | 148.00 | 150.00 | 100.00 | 93788.00 | 3.38 | 109.80 |
| EP76 | Responder | Validation | Post-treatment | 125.00 | 1.94 | 348.75 | 4.68 | 9.38 | 3.15 | 54.60 | 258.00 | 29.40 |
| EP77 | Non-responder | Validation | Post-treatment | 209.72 | 2.93 | 442.51 | 221.00 | 142.00 | 44.80 | 28.10 | 3.04 | 17.00 |
| EP78 | Responder | Validation | Post-treatment | 202.35 | 2.26 | 388.52 | 5.90 | 5.93 | 6.78 | 8.33 | 7.17 | 8.00 |
| EP79 | Responder | Validation | Post-treatment | 193.18 | 1.84 | 312.95 | 2.69 | 7.28 | 7.50 | 11.39 | 6.56 | 2.20 |
| EP80 | Responder | Validation | Post-treatment | 160.53 | 3.18 | 388.47 | 160.00 | 9.03 | 9.28 | 2.89 | 3.28 | 32.38 |
| EP81 | Non-responder | Validation | Post-treatment | 368.57 | 12.23 | 1577.49 | 3.96 | 1.10 | 15.00 | 6.48 | 2.34 | 159.81 |
| EP82 | Responder | Validation | Post-treatment | 135.98 | 3.32 | 741.07 | 51.00 | 7.76 | 11.50 | 109.00 | 2.87 | 35.30 |
| EP83 | Non-responder | Validation | Post-treatment | 282.46 | 14.04 | 2259.65 | 2.25 | 1.27 | 28.20 | 12.05 | 10.04 | 62.14 |
| EP84 | Non-responder | Validation | Post-treatment | 117.59 | 3.62 | 459.79 | 1.90 | 3.87 | 12.00 | 7.00 | 92.50 | 11.80 |
| EP85 | Non-responder | Validation | Post-treatment | 77.53 | 0.93 | 162.82 | 8.68 | 5.57 | 62.20 | 19.70 | 3.18 | 58.00 |
| EP86 | Non-responder | Validation | Post-treatment | 106.45 | 1.55 | 409.84 | 8491.00 | 4.94 | 26.00 | 2.00 | 27.50 | 43.00 |
| EP87 | Responder | Validation | Post-treatment | 123.64 | 2.41 | 327.64 | 2.84 | 9.44 | 29.20 | 29.10 | 3.99 | 25.40 |
| EP89 | Responder | Validation | Post-treatment | 354.43 | 5.71 | 1598.48 | 3.44 | 2.38 | 9.30 | 5.34 | 7.38 | 61.97 |
| EP90 | Responder | Validation | Post-treatment | 196.00 | 3.83 | 750.68 | 134.00 | 204.00 | 2.89 | 208.00 | 2.96 | 55.60 |
| EP91 | Responder | Validation | Post-treatment | 93.75 | 13.23 | 794.06 | 3.03 | 1.13 | 5.10 | 7.79 | 1.24 | 3.13 |
| EP92 | Non-responder | Validation | Post-treatment | 81.66 | 2.05 | 282.53 | 3.16 | 12.40 | 4.90 | 3.08 | 0.61 | 37.53 |
| EP93 | Responder | Validation | Post-treatment | 138.78 | 1.41 | 288.65 | 7.11 | 4.34 | 7.90 | 8.09 | 2.10 | 10.16 |
| EP94 | Responder | Validation | Post-treatment | 133.98 | 8.68 | 1197.79 | 4.03 | 4.90 | 6.40 | 3.71 | 3.88 | 13.06 |
| EP95 | Responder | Validation | Post-treatment | 306.06 | 5.06 | 1533.36 | 0.95 | 1.03 | 7.40 | 29.18 | 1.60 | 58.92 |
| EP96 | Non-responder | Validation | Post-treatment | 492.86 | 12.36 | 1705.29 | 34.16 | 54.32 | 38.20 | 1000.00 | 2.03 | 45.02 |
| EP97 | Non-responder | Validation | Post-treatment | 104.15 | 2.61 | 360.36 | 2.09 | 1.76 | 11.80 | 69.10 | 3.79 | 21.60 |
| EP99 | Responder | Validation | Post-treatment | 115.50 | 2.05 | 306.09 | 3.06 | 9.04 | 11.00 | 16.20 | 3.28 | 31.00 |

*PLR, platelet-to-lymphocyte ratio;

†NLR, neutrophil-to-lymphocyte ratio;

††SII, systemic immune-inflammation index.

**Supplementary Table 4. Differential expression of small RNAs between responders and non-responders among aGC patients in baseline plasma samples.**

| **NO.** | **Small RNA** | **Type** | **CPM** | | **Fold change (R/NR)** | ***p* value** |
| --- | --- | --- | --- | --- | --- | --- |
|  |  |  | **R** | **NR** |  |  |
| 1 | hsa-miR-181d-5p | miRNA | 152.13 | 640.54 | 0.24 | 0.046 |
| 2 | hsa-miR-3916 | miRNA | 212.60 | 134.35 | 1.58 | 0.020 |
| 3 | hsa-miR-1260b | miRNA | 211.93 | 116.17 | 1.82 | 0.025 |

**Supplementary Table 5. Differential expression of small RNAs between pre- and post-treatment among aGC patients in paired plasma samples.**

| **NO.** | **Small RNAs** | **Type** | **CPM** | | **Fold change (post / pre)** | ***p* value** |
| --- | --- | --- | --- | --- | --- | --- |
|  |  |  | **pre** | **post** |  |  |
| 1 | hsa-miR-144-3p | miRNA | 87.45 | 34.01 | 0.39 | 0.013 |
| 2 | hsa-miR-204-5p | miRNA | 222.44 | 102.42 | 0.46 | 0.018 |
| 3 | hsa-miR-320c | miRNA | 284.37 | 98.15 | 0.35 | 0.021 |
| 4 | hsa-miR-328-3p | miRNA | 161.60 | 34.44 | 0.21 | 0.019 |
| 5 | hsa-miR-942-5p | miRNA | 242.83 | 35.57 | 0.15 | < 0.001 |
| 6 | hsa-miR-9-5p | miRNA | 226.76 | 70.91 | 0.31 | 0.019 |
| 7 | mature-tRNA-Arg-CCT | mature-tRNA | 263.07 | 157.17 | 0.60 | 0.026 |
| 8 | mature-tRNA-His-GTG | mature-tRNA | 938.79 | 541.32 | 0.58 | 0.020 |
| 9 | mature-tRNA-Trp-CCA | mature-tRNA | 935.03 | 265.93 | 0.28 | 0.021 |
| 10 | 5.8S-rRNA | rsRNA | 2786.04 | 4255.11 | 1.53 | < 0.001 |
| 11 | hsa-let-7a-3p | miRNA | 19.23 | 52.89 | 2.75 | 0.033 |
| 12 | hsa-miR-122-5p | miRNA | 6560.24 | 10377.41 | 1.58 | 0.009 |
| 13 | hsa-miR-143-3p | miRNA | 159.92 | 585.58 | 3.66 | < 0.001 |
| 14 | hsa-miR-15b-5p | miRNA | 1402.08 | 2178.33 | 1.55 | 0.009 |
| 15 | hsa-miR-16-5p | miRNA | 3095.76 | 5401.30 | 1.74 | 0.014 |
| 16 | hsa-miR-185-5p | miRNA | 484.29 | 1101.10 | 2.27 | < 0.001 |
| 17 | hsa-miR-26b-5p | miRNA | 323.66 | 890.40 | 2.75 | 0.003 |
| 18 | mature-tRNA-Glu-TTC | mature-tRNA | 729.21 | 1138.09 | 1.56 | 0.009 |

**Supplementary Table 6. Differential plasma small RNA levels between pre- and post-treatment in responders and non-responders of aGC patients in the discovery cohort.**

| **NO.** | **Small RNAs** | **Type** | **Responder** | | | |  | **Non-responder** | | | |  |
| --- | --- | --- | --- | --- | --- | --- | --- | --- | --- | --- | --- | --- |
|  |  |  | **CPM** | | **Fold change (post/pre)** | ***p* value** | **Change^*^** | **CPM** | | **Fold change (post/pre)** | ***p* value** | **Change^*^** |
|  |  |  | **pre** | **post** |  |  |  | **pre** | **post** |  |  |  |
| 1 | hsa-miR-144-3p | miRNA | 65.12 | 37.35 | 0.57 | 0.076 | Not | 138.06 | 26.45 | 0.19 | 0.050 | Not |
| 2 | hsa-miR-204-5p | miRNA | 219.23 | 94.48 | 0.43 | 0.039 | **Down** | 229.70 | 120.43 | 0.52 | 0.174 | Not |
| 3 | hsa-miR-320c | miRNA | 86.98 | 8.76 | 0.10 | 0.006 | **Down** | 731.78 | 300.79 | 0.41 | 0.653 | Not |
| 4 | hsa-miR-328-3p | miRNA | 217.54 | 26.63 | 0.12 | 0.005 | **Down** | 34.81 | 52.13 | 1.50 | 0.624 | Not |
| 5 | hsa-miR-942-5p | miRNA | 279.87 | 23.33 | 0.08 | 0.001 | **Down** | 158.89 | 63.33 | 0.40 | 0.233 | Not |
| 6 | hsa-miR-9-5p | miRNA | 179.55 | 62.01 | 0.35 | 0.035 | **Down** | 333.77 | 91.07 | 0.27 | 0.250 | Not |
| 7 | mature-tRNA-Arg-CCT | mature-tRNA | 226.44 | 178.47 | 0.79 | 0.113 | Not | 346.11 | 108.88 | 0.31 | 0.174 | Not |
| 8 | mature-tRNA-His-GTG | mature-tRNA | 802.39 | 542.21 | 0.68 | 0.056 | Not | 1247.97 | 539.31 | 0.43 | 0.148 | Not |
| 9 | mature-tRNA-Trp-CCA | mature-tRNA | 1192.87 | 292.48 | 0.25 | 0.037 | **Down** | 350.61 | 205.76 | 0.59 | 0.412 | Not |
| 10 | 5.8S-rRNA | rsRNA | 2911.80 | 4707.23 | 1.62 | < 0.001 | **Up** | 2500.97 | 3230.30 | 1.29 | 0.325 | Not |
| 11 | hsa-let-7a-3p | miRNA | 15.75 | 62.21 | 3.95 | 0.044 | **Up** | 27.12 | 31.76 | 1.17 | 0.389 | Not |
| 12 | hsa-miR-122-5p | miRNA | 5604.38 | 10498.21 | 1.87 | 0.010 | **Up** | 8726.86 | 10103.61 | 1.16 | 0.412 | Not |
| 13 | hsa-miR-143-3p | miRNA | 108.20 | 470.31 | 4.35 | < 0.001 | **Up** | 277.13 | 846.87 | 3.06 | 0.010 | **Up** |
| 14 | hsa-miR-15b-5p | miRNA | 1333.73 | 1953.41 | 1.46 | 0.053 | Not | 1557.02 | 2688.16 | 1.73 | 0.061 | Not |
| 15 | hsa-miR-16-5p | miRNA | 2695.22 | 4720.74 | 1.75 | 0.180 | Not | 4003.66 | 6943.89 | 1.73 | 0.037 | **Up** |
| 16 | hsa-miR-185-5p | miRNA | 404.48 | 1075.33 | 2.66 | 0.000 | **Up** | 665.17 | 1159.51 | 1.74 | 0.081 | Not |
| 17 | hsa-miR-26b-5p | miRNA | 240.41 | 947.67 | 3.94 | 0.007 | **Up** | 512.34 | 760.60 | 1.48 | 0.233 | Not |
| 18 | mature-tRNA-Glu-TTC | mature-tRNA | 438.10 | 1216.17 | 2.78 | 0.005 | **Up** | 1389.07 | 961.11 | 0.69 | 0.838 | Not |

*UP, up-regulated; DOWN, down-regulated; NOT, not significant.
